# Metformin potentiates nephrotoxicity by promoting NETosis in response to renal ferroptosis

**DOI:** 10.1038/s41421-023-00595-3

**Published:** 2023-10-17

**Authors:** Zhaoxian Cai, Xiaotian Wu, Zijun Song, Shumin Sun, Yunxing Su, Tianyi Wang, Xihao Cheng, Yingying Yu, Chao Yu, En Chen, Wenteng Chen, Yongping Yu, Andreas Linkermann, Junxia Min, Fudi Wang

**Affiliations:** 1 The Second Affiliated Hospital, The First Affiliated Hospital, School of Public Health, Institute of Translational Medicine, Cancer Center, Zhejiang University School of Medicine, Hangzhou, Zhejiang China; 2 The First Affiliated Hospital, Basic Medical Sciences, School of Public Health, Hengyang Medical School, University of South China, Hengyang, Hunan China; 3 College of Pharmaceutical Science, Zhejiang University, Hangzhou, Zhejiang China; 4 Division of Nephrology, Department of Internal Medicine III, University Hospital Carl Gustav Carus at the Technische Universität Dresden, Dresden, Germany; 5 Division of Nephrology, Department of Medicine, Albert Einstein College of Medicine, Bronx, NY USA

**Keywords:** Mechanisms of disease, Entosis

## Abstract

Given the rapidly aging population, aging-related diseases are becoming an excessive burden on the global healthcare system. Metformin has been shown to be beneficial to many age-related disorders, as well as increase lifespan in preclinical animal models. During the aging process, kidney function progressively declines. Currently, whether and how metformin protects the kidney remains unclear. In this study, among longevity drugs, including metformin, nicotinamide, resveratrol, rapamycin, and senolytics, we unexpectedly found that metformin, even at low doses, exacerbated experimentally-induced acute kidney injury (AKI) and increased mortality in mice. By single-cell transcriptomics analysis, we found that death of renal parenchymal cells together with an expansion of neutrophils occurs upon metformin treatment after AKI. We identified programmed cell death by ferroptosis in renal parenchymal cells and blocking ferroptosis, or depleting neutrophils protects against metformin-induced nephrotoxicity. Mechanistically, upon induction of AKI, ferroptosis in renal parenchymal cells initiates the migration of neutrophils to the site of injury via the surface receptor CXCR4–bound to metformin–iron–NGAL complex, which results in NETosis aggravated AKI. Finally, we demonstrated that reducing iron showed protective effects on kidney injury, which supports the notion that iron plays an important role in metformin-triggered AKI. Taken together, these findings delineate a novel mechanism underlying metformin-aggravated nephropathy and highlight the mechanistic relationship between iron, ferroptosis, and NETosis in the resulting AKI.

## Introduction

By 2050, the older segment of the world population is expected to number approximately 2 billion individuals^1^. However, aging is the greatest risk factor for the development of most common chronic human diseases^2^. An increasing number of strategies have been identified to therapeutically target fundamental aging, such as the rejuvenating effect of young blood on aging-related memory loss^3^, impaired motor ability^4,5^ and organ dysfunction^6^, as well as with several longevity drugs now being tested to improve lifespan and healthspan^7^. Previous studies have shown that metformin (Met)^8^, nicotinamide (NA)^9^, resveratrol (Res)^10^, rapamycin (Rap)^11^, and senolytics (Sen)^12^ could improve both lifespan and healthspan and thus have become the focus of longevity medicine.

Notably, more than 10% of adults globally have kidney diseases^13^. Among them, acute kidney injury (AKI) is the most common cause of overall organ dysfunction and is associated with high morbidity and mortality rates among critically ill patients^14^. And during the ongoing coronavirus pandemic, several studies found that patients hospitalized with COVID-19 have a high incidence of AKI^15^. In addition, patients with AKI have a high risk of developing chronic kidney diseases (CKD) and kidney failure, as well as other types of organ failure^16^. Despite a growing body of evidence indicating that the death of tubular epithelial cells plays a pathogenetic role in AKI^17^, the precise mechanisms of AKI remain poorly understood. Similarly, the lack of specific drugs to treat various kidney diseases remains an important unmet clinical need, and in the field of gerontology, the prevention and treatment of kidney disease is an urgent issue given its role as a key risk factor for mortality.

Metformin, a biguanide derivative, was first described nearly a century ago and is currently the first-line treatment for type 2 diabetes^18^. Recently, metformin has been shown to have beneficial effects on a variety of diseases and conditions^19^, including cancer^20^, obesity^21^, liver diseases^22^, and cardiovascular diseases^23^. Given these beneficial effects, metformin has been recommended for people without diabetes. In clinical practice, metformin is generally well tolerated^24^. However, the activity of metformin in AKI is still incompletely understood. To address this question, and to find lifespan-extending drugs that might have benefits for kidney disease, we investigated the effect of several longevity drugs on the progression of AKI in relevant mouse models with a focus on metformin.

## Results

### Metformin aggravates AKI in mouse models in a dose-dependent manner

**Fig. 1:**
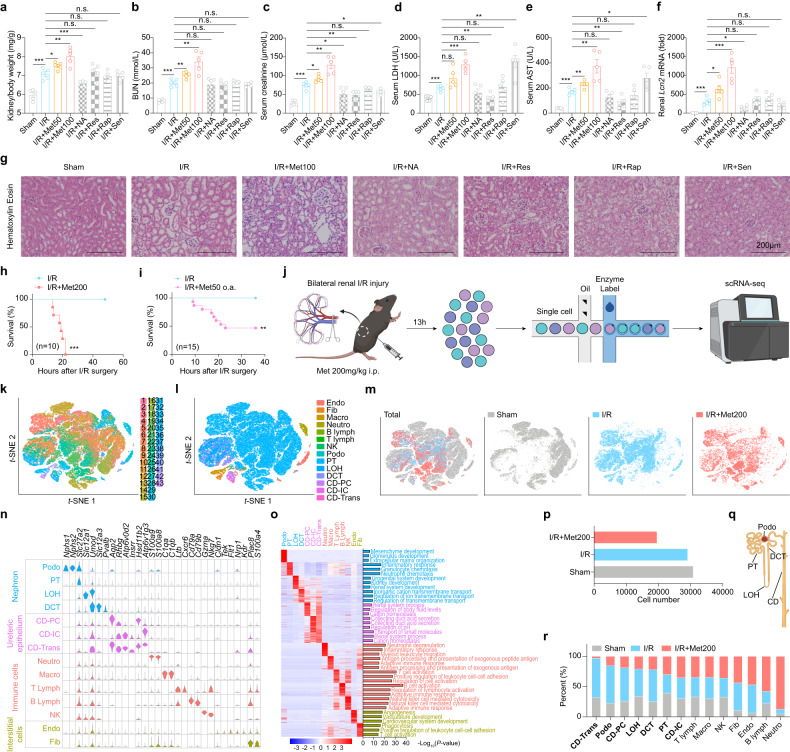
Metformin induces renal parenchymal cell death to exacerbate acute kidney injury in a dose-dependent manner. **a** Summary of the kidney to body weight ratio measured in the Sham, I/R, I/R+Met50 (50 mg/kg i.p.), I/R+Met100 (100 mg/kg i.p.), and I/R + NA (50 mg/kg i.p.), I/R+Res (100 mg/kg i.p.), I/R+Rap (10 mg/kg i.p.) and I/R+Sen (dasatinib 5 mg/kg i.p. and quercetin 50 mg/kg i.p.) mice 24 h after I/R. **b**–**e** Summary of serum BUN (**b**), creatinine (**c**), LDH (**d**), and AST (**e**) measured in the indicated mice. **f** Summary of renal *Lcn2* mRNA level measured in the indicated mice, expressed relative to control. **g** Representative Hematoxylin and Eosin (H&E)-stained kidney sections from indicated mice. **h** Kaplan–Meier survival curves of mice in the I/R and I/R+Met200 (200 mg/kg i.p.) groups (*n* = 10 mice per group). **i**. Kaplan–Meier survival curves of mice in the I/R and I/R+Met50 (50 mM o.a.) groups (*n* = 10 mice per group). **j** Overview of the experimental design, depicting the strategy for isolating renal cells and performing scRNA-seq. **k** T-distributed stochastic neighbor embedding (*t*-SNE) reveals clustering of 79,224 renal cells obtained from mice in sham group (*n* = 2), I/R group (*n* = 2), and I/R+Met200 group (*n* = 2). **l**. *t*-SNE reveals 14 distinct cell types in the kidney. **m** Annotation of renal cells in sham, I/R, and I/R+Met200 groups. **n** Violin plots showing the expression of the indicated marker genes in each cell type. **o** Left: heatmap of the differential expression of the clustered marker genes measured in each cell type. Right: GO enrichment terms for the various cell types. **p** Summary of the cell numbers included in the scRNA-seq analysis for mice in sham group (*n* = 2), I/R group (*n* = 2), and I/R+Met200 group (*n* = 2). **q** Summary of the percentage of the indicated cell types in the indicated groups. **r** Schematic diagram of renal parenchymal cell types. **P < 0.01, ***P < 0.001 and n.s., not significant (One-way ANOVA). Survival curve comparison was analyzed by a log-rank Mantel-Cox test. In this and subsequent figures, summary data are represented as the mean ± SEM. See also Supplementary Figs. S1–S4.

To explore whether different longevity drugs (Supplementary Fig. S1) can be a candidate for treating AKI, we treated mice with AKI induced by renal ischemia-reperfusion (I/R) with different drugs alone. Notably, nicotinamide, resveratrol, rapamycin, and senolytics, but not metformin, showed some to no protective effects on AKI based on various readouts of renal health and function (Fig. 1a–g). Notably, based on previous studies with respect to the effective dose of metformin in mice^25^, we purposely chose low (50 mg/kg and 100 mg/kg) and high (200 mg/kg) doses of metformin for the study. However, we found that even low doses of metformin led to the exacerbation of I/R-induced AKI (Fig. 1a–f). In addition, a higher dose of metformin (200 mg/kg) resulted in 100% lethality of I/R mice while the untreated I/R mice showed 100% survival (Fig. 1h).

To explore the effect of chronic metformin treatment before AKI occurred, we orally treated mice with lower dose 50 mM metformin for 7 days before renal I/R surgery, and surprisingly we found that metformin-aggravated I/R-induced AKI, inducing > 50% death within 24 h (Fig. 1i). Furthermore, higher dose of metformin (200 mg/kg) caused 100% lethality in a rhabdomyolysis-induced model of AKI (Supplementary Fig. S2a, b). And like in the I/R-induced AKI model, lower doses of metformin exacerbated various renal injury-related parameters in the rhabdomyolysis-induced AKI mouse model (Supplementary Fig. S2c–k). Moreover, an accumulation of metformin in patients with kidney failure can cause lactic acidosis, a rare yet potentially fatal condition^26^. Notably, however, metformin at 100 mg/kg had no significant effects on serum lactic acid (Supplementary Fig. S3a) or glucose levels (Supplementary Fig. S3b) in the I/R-induced AKI model. Similarly, metformin (100 mg/kg) had no influence on serum glucose and lactic acid levels in the rhabdomyolysis-induced AKI mouse model (Supplementary Fig. S2i, j). Notably, it is suggested that the effects of metformin at those doses on AKI are not due to either an accumulation of lactic acid or hypoglycemia.

To test human relevance, we explored the association between metformin and kidney injury or diseases from cohort studies. We analyzed the data of 49 cohorts and found that although the overall OR < 1 (Supplementary Fig. S4a, b and Table S1), it could still be found that 14 cohorts showed that metformin was significantly associated with severe kidney injury or diseases (Supplementary Fig. S4c).

### Single-cell profiling of kidney cells obtained from metformin-treated I/R mice

To explore the mechanism that may explain the potential nephrotoxicity of metformin, we next performed single-cell RNA sequencing (scRNA-seq) with cells isolated from the kidneys of sham-treated mice, mice with I/R-induced AKI, and high-dose metformin-treated I/R mice (Fig. 1j). Our scRNA-seq analysis revealed 43 clusters based on transcriptomes identified using T-distributed stochastic neighbor embedding (*t*-SNE) visualization (Fig. 1k). Based on the expression of cell type-specific marker genes^27^, these 43 clusters were then classified into 14 distinct cell types and these cell types were present in each treatment group (Fig. 1l–n; Supplementary Table S2). Further gene ontology (GO) and gene set enrichment analysis (GSEA) of each cell type revealed corresponding cell type-specific pathways (Fig. 1o).

### Death of renal parenchymal cells contributes to metformin-induced nephrotoxicity

**Fig. 2:**
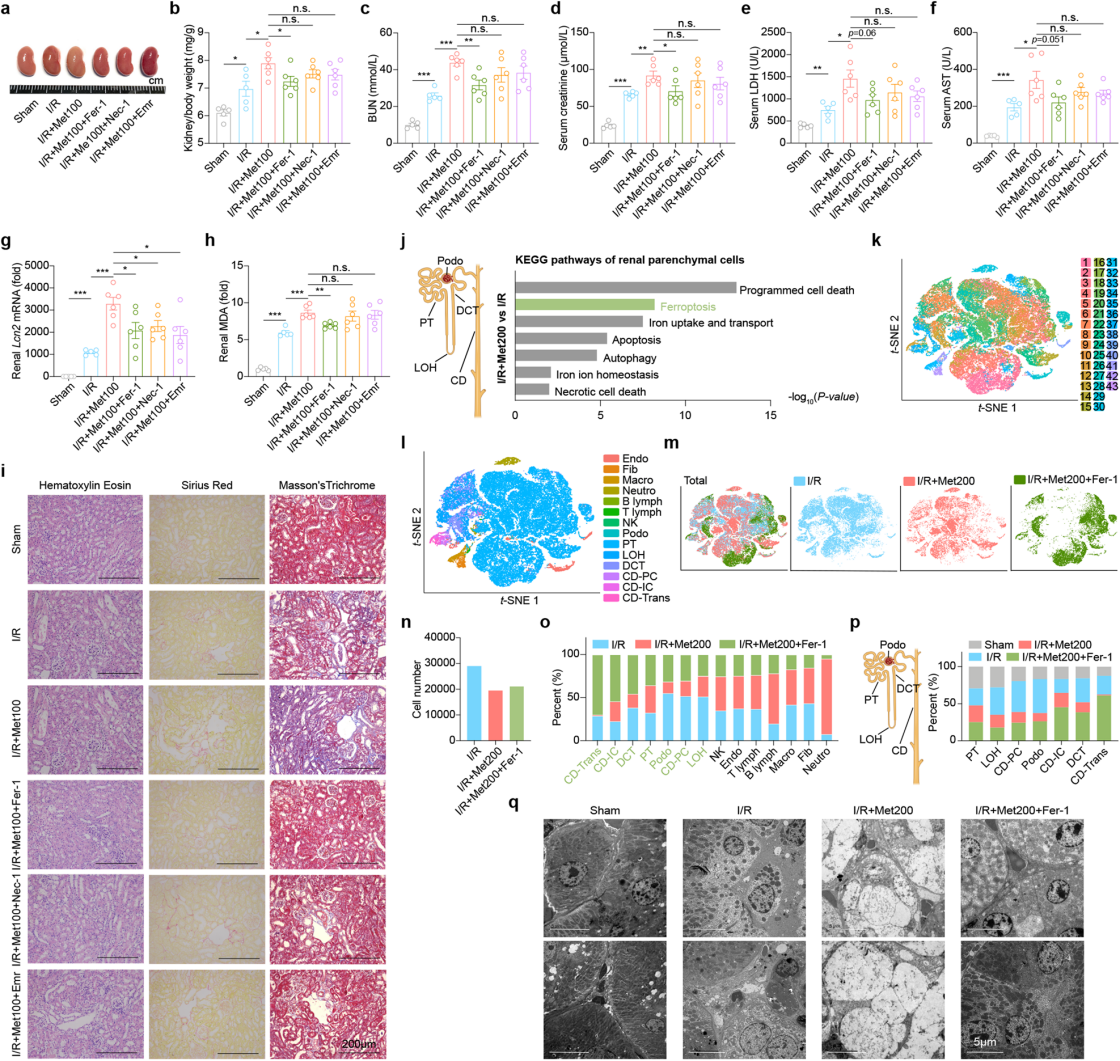
Renal parenchymal cell ferroptosis is critical for metformin-aggravated nephrotoxicity. **a** Representative image of kidneys removed from mice in the indicated groups. Where indicated, the mice received metformin (100 mg/kg), Fer-1 (1 mg/kg), Nec-1 (1 mg/kg), or Emr (2.5 mg/kg). **b** Summary of the kidney to body weight ratio measured in the indicated mice. **c**–**f** Summary of serum BUN (**c**), creatinine (**d**), LDH (**e**), and AST (**f**) measured in the indicated mice. **g** Summary of renal *Lcn2* mRNA levels measured in the indicated mice. **h** Summary of renal MDA levels measured in the indicated mice. **i** Representative H&E-stained, Sirius Red-stained, and Masson’s Trichrome-stained kidney sections from the indicated mice. **j** Summary of significantly differential pathways in the indicated parenchymal cell types of I/R+Met200 vs I/R. **k** *t*-SNE reveals clustering of 69,608 renal cells obtained from mice in the I/R group (*n* = 2), I/R+Met200 group (*n* = 2), and I/R+Met200+Fer-1 group (*n* = 2). **l**. *t*-SNE reveals 14 distinct cell types in the kidney. **m** Annotation of renal cells in I/R, I/R+Met200 and I/R+Met200+Fer-1 groups. **n** Summary of the number of cells included in the scRNA-seq analysis for the indicated groups (*n* = 2 mice per group), expressed relative to the Sham. **o** Summary of the percentages of the indicated cell types in the indicated groups. **p** Summary of the percentage of the indicated renal parenchymal cell types in 4 groups shown at the left. **q** Electron micrographs showing the cellular morphology of renal tubular epithelial cells in the indicated groups. *P < 0.05, **P < 0.01, ***P < 0.001, and n.s., not significant (One-way ANOVA). See also Supplementary Figs. S5–S8.

We found that the metformin-treated I/R mice had approximately one-third fewer cells compared to the other two groups (Fig. 1p). Interestingly, we also found that the metformin-treated I/R mice had significantly fewer renal parenchymal cells compared to the I/R group (Fig. 1q, r). Several forms of regulated cell death have been observed in various models of AKI, including apoptosis^28^, necroptosis^29^, and ferroptosis^30,31^. Previous studies^32^ found that inhibiting specific forms of cell death can partially protect renal function during AKI, suggesting that these various forms of cell death play a complex, intertwined role in the pathogenesis of AKI. We tested the effect of inhibiting these three forms of cell death using emricasan (Emr), necrostatin-1 (Nec-1), ferrostain-1 (Fer-1)^33^, respectively, on lower dose of metformin-induced exacerbation of AKI. Overall, when examining the effects on renal hypertrophy, function, and damage (Fig. 2a–h) we found there was a protective effect for all three inhibitors with respect to a marker of kidney injury — *Lcn2* mRNA levels (Fig. 2g) — in metformin-treated I/R-induced AKI mice. But for all the other parameters examined only the inhibition of ferroptosis resulted in significant reduction in *Lcn2* expression compared to the metformin-treated I/R mice, though none of the parameters were below the sham readouts. Consistent with these results, Fer-1, Nec-1, and Emr also improved tissue morphology and reduced fibrosis in the renal cortex of metformin-treated I/R mice (Fig. 2i). Notably, additional ferroptosis inhibitor, liproxstatin-1 (Lip-1) and necroptosis inhibitor, necrostatin 2 reacmate (Nec-1s) also showed protective effect on metformin nephrotoxicity (Supplementary Fig. S5a–f). Similar results were obtained using Fer-1, Nec-1, and Emr in metformin-treated mice with rhabdomyolysis-induced AKI mice (Supplementary Fig. S6a–i). The above results suggest that metformin-aggravated nephrotoxicity causes multiple forms of programmed cell death in renal parenchymal cells. And by KEGG pathway analysis of renal parenchymal cells, we found that multiple cell death pathways were significantly changed, with the most changed being ferroptosis (Fig. 2j).

As multiple types of cell death participated in the metformin-aggravated nephrotoxicity, we decided to focus on ferroptosis as the representative form of cell death to analyze the detailed effect of inhibitors as it was the most changed, as well as that to date it is the least studied form of cell death in AKI. We found that Fer-1 also provided significant benefits in I/R mice given a higher dose of metformin (200 mg/kg), as revealed by the kidney to body weight ratio (Supplementary Fig. S7a), serological indicators (Supplementary Fig. S7b–d), and renal morphology (Supplementary Fig. S7e) measured 13 h after I/R, without affecting other organs (Supplementary Fig. S8). To identify the targeted cell types, we used scRNA-seq to analyze the kidney cells in the groups. Using the same gene expression markers in Fig. 1n, the 43 clusters (Fig. 2k) were again classified into 14 cell types (Fig. 2l; Supplementary Table S2). Based on *t*-SNE dimensionality reduction analysis, we visualized the cell distribution (Fig. 2m) and cell numbers (Fig. 2n, o) in all three groups. Notably, we found that the renal parenchymal cells were rescued by Fer-1 (Fig. 2p).

Proximal tubular epithelial (PT) cells are the most abundant cells in the renal parenchyma. We found that metformin caused major morphological changes in PT cells in the I/R group, including decreased or absent mitochondrial crests, as well as rupture and shrinkage of the outer mitochondrial membrane; moreover, these changes were largely prevented by treating the mice with Fer-1 in higher dose of metformin-aggravated I/R-induced AKI (Fig. 2q).

### Decreased iron levels ameliorate metformin-induced nephrotoxicity

**Fig. 3:**
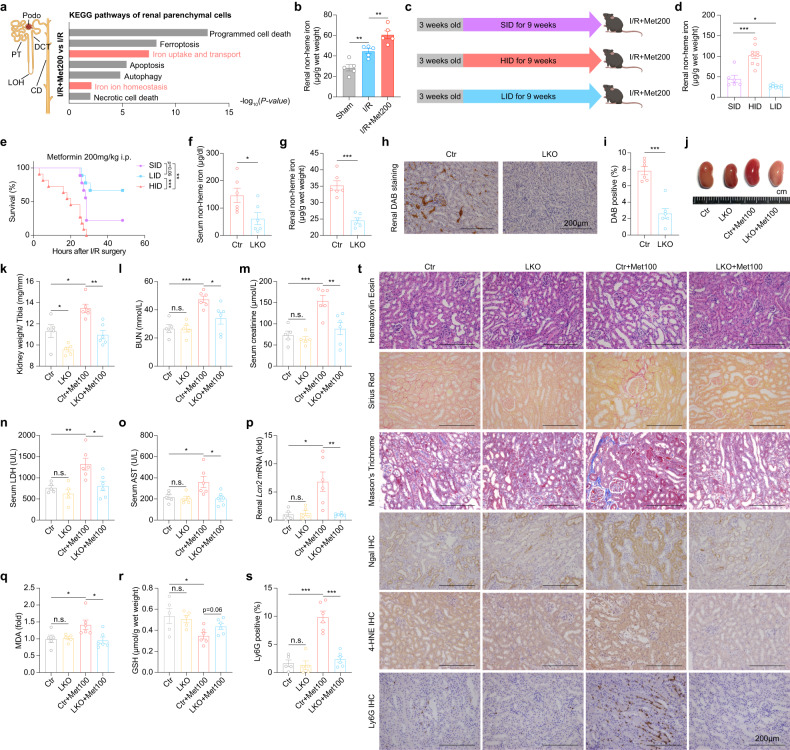
Decreased iron level alleviates metformin-induced nephrotoxicity in the context of AKI. **a** Summary of significantly differentially pathways in the indicated parenchymal cell types of I/R+Met200 vs I/R. **b** Summary of renal non-heme iron levels measured in the Sham mice, I/R mice, and I/R+Met200 mice. **c** At 3 weeks of age, mice were fed a standard-iron diet (SID), high-iron diet (HID), or low-iron diet (LID) for 9 weeks, then subjected to I/R+Met200. **d** Summary of renal non-heme iron levels measured in the indicated mice. **e** Kaplan–Meier survival curves of the indicated mice (*n* = 11, 9, and 9 mice in the HID, SID, and LID groups, respectively). **f** Summary of serum iron levels measured in adult *Tmprss6*^*flox/flox*^ and *Tmprss6*^*Alb/Alb*^ mice. **g** Summary of renal non-heme iron levels measured in the indicated mice. **h** Representative DAB-stained kidney sections from the indicated mice. **i** Summary of the percentages of DAB-positive area measured in DAB-stained kidney sections. **j** Representative image of kidneys in the indicated mice. **k** Summary of the ratio between kidney weight and tibia length measured in the indicated mice. **l**–**o** Summary of serum BUN (**l**), creatinine (**m**), LDH (**n**), and AST (**o**) measured in the indicated mice. **p** Summary of renal *Lcn2* mRNA levels measured in the indicated mice, expressed relative to control. **q** Summary of renal MDA levels measured in the indicated mice. **r** Summary of renal reductive GSH levels measured in the indicated mice. **s** Summary of the percentages of Ly6G-positive area measured in immunostained kidney sections. **t** Representative H&E-stained, Sirius Red-stained, Masson’s Trichrome-stained, NGAL-stained, 4-HNE-stained and Ly6G-stained kidney sections from the indicated mice. *P < 0.05, **P < 0.01, ***P < 0.001 and n.s., not significant (one-way ANOVA). Survival curve comparison was analyzed by a log-rank Mantel-Cox test. In this and subsequent figures, summary data are represented as the mean ± SEM. See also Supplementary Figs. S9 and S10.

Among the various pathways affected, we noted that the iron uptake and transport pathway and the iron homeostasis pathway were significantly altered in the I/R+Met200 group (Fig. 3a), as iron transport and lipid peroxidation are key drivers of ferroptosis^31,34^. We also found that the renal non-heme iron level increased in the I/R+Met200 groups, progressively (Fig. 3b). Thus, we fed mice a high-iron diet (HID), a standard-iron diet, (SID), or a low-iron diet (LID) for 9 weeks and then induced I/R together with metformin treatment (Fig. 3c, d; Supplementary Fig. S9). We found that compared to SID-fed mice, the HID-fed mice had significantly increased mortality, while the LID-fed mice had significantly prolonged survival (Fig. 3e).

The enzyme TMPRSS6 plays a critical role in mediating iron homeostasis, and mutations in human TMPRSS6 as well as global *Tmprss6*-deficient mice have been linked to iron-refractory iron deficiency anemia (IRIDA)^35,36^. To test whether low iron can protect against metformin-induced nephrotoxicity and to exclude the function of renal *Tmprss6* itself, we generated a liver-specific *Tmprss6* knockout mouse (LKO, *Tmprss6*^*Alb/Alb*^), instead of global *Tmprss6* knockout mice (Supplementary Fig. S10a, b). Compared to control mice, LKO mice had significantly reduced iron levels in multiple organs (Supplementary Fig. S10c), as well as reduced serum iron levels (Fig. 3f) and typical features of iron-deficiency anemia (Supplementary Fig. S10d). In addition, DAB staining showed that renal iron levels were significantly lower in the nephrons of LKO mice compared to control (Ctr) mice (Fig. 3g–i). In addition, LKO mice had iron deficiency-associated anemia (Supplementary Fig. S10e–h) with no inflammation (Supplementary Fig. S10i). Notably, LKO mice were less sensitive to the effects of metformin on I/R-induced AKI, as reflected by improved renal hypertrophy (Fig. 3j, k), lower serum BUN, creatinine, LDH, and AST levels (Fig. 3l–o), as well as lower *Lcn2* mRNA level (Fig. 3p). Also, LKO mice showed lower renal MDA (Fig. 3q), higher renal GSH/GSSG (Fig. 3r) and lower renal 4-HNE levels (Fig. 3t) in the I/R+Met100 condition, compared to Ctr+Met100 mice, indicating LKO mice were protected from ferroptosis in metformin-aggravated nephrotoxicity. Finally, loss of hepatic *Tmprss6* protected against the histological changes caused by I/R+Met (Fig. 3t).

### Renal accumulation of neutrophils and NETosis play important role in metformin-aggravated nephrotoxicity

**Fig. 4:**
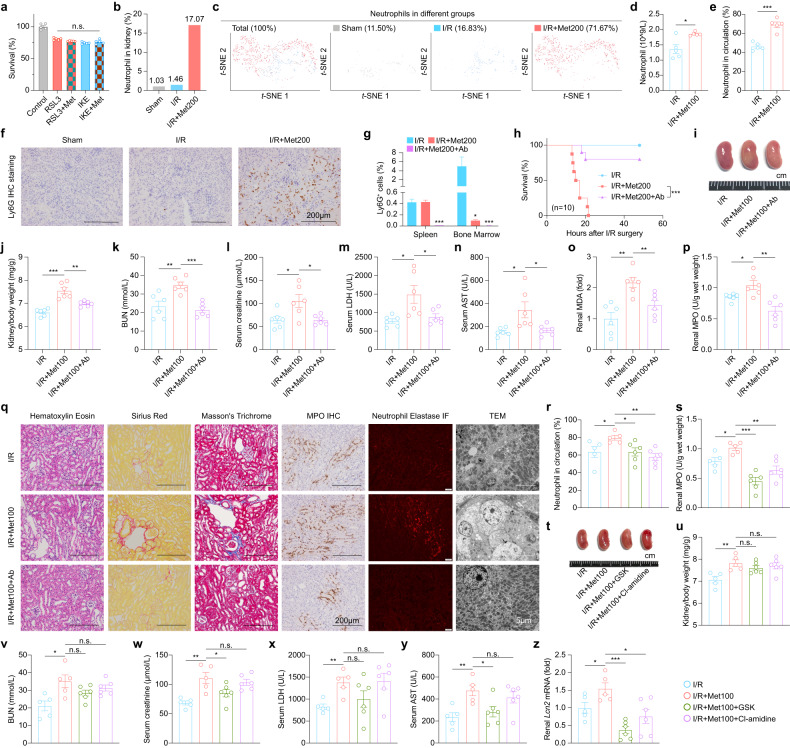
Neutrophil-released NETosis plays a key role in driving metformin nephrotoxicity. **a** Survival of the RSL3 (5 µM) or IKE (10 µM) induced ferroptotic 293T cells intervened with metformin (200 µM) for 24 h. **b** Summary of the percentages of neutrophils in sham group (*n* = 2), I/R group (*n* = 2), and I/R+Met200 group (*n* = 2). **c** *t*-SNE reveals neutrophils cluster in the indicated groups. **d** Summary of the numbers of neutrophils in circulation in I/R mice and I/R+Met100 mice. **e** Summary of the percentages of neutrophils in circulation in I/R mice and I/R+Met100 mice. **f** Representative Ly6G immunohistochemistry (IHC)-stained kidney sections from the indicated groups. **g** Flow cytometry analysis of the percentages of neutrophils measured in the spleen and bone marrow of the indicated mice; where indicated, metformin and the anti-Ly6G antibody were administered by i.p. injection at 200 mg/kg and 200 µg per mouse, respectively. **h** Kaplan–Meier survival curves of the indicated mice (*n* = 10 mice per group). **i** Representative image of kidneys in the indicated groups; where indicated, metformin and anti-Ly6G was administered at 100 mg/kg and 200 µg per mouse, respectively. **j** Summary of the kidney to body weight ratio measured in the indicated mice. **k**–**n** Summary of serum BUN (**k**), creatinine (**l**), LDH (**m**), and AST (**n**) measured in the indicated mice. **o** Summary of renal MDA levels in the indicated mice. **p** Summary of renal myeloperoxidase (MPO) levels in the indicated mice. **q** Representative H&E-stained, Sirius Red-stained, Masson’s Trichrome-stained, MPO IHC-stained, Neutrophil Elastase IF-stained, and electron micrographs showing kidney sections from the indicated mice. **r** Summary of the percentages of neutrophils in circulation in I/R, I/R+Met100, I/R+Met100+GSK, and I/R+Met100+Cl-amidine mice. **s** Summary of renal MPO level in the indicated mice. **t** Representative image of kidneys in the indicated groups. **u** Summary of the kidney to body weight ratio measured in the indicated mice. **v**–**y** Summary of serum BUN (**v**), creatinine (**w**), LDH (**x**), and AST (**y**) measured in the indicated mice. **z** Summary of renal *Lcn2* mRNA levels in the indicated mice, expressed relative to control. *P < 0.05, **P < 0.01, ***P < 0.001 (one-way ANOVA). Survival curve comparison was analyzed by a log-rank Mantel-Cox test. In this and subsequent figures, summary data are represented as the mean ± SEM. See also Supplementary Figs. S11, S12.

Although we found there was no obvious toxicity effect of the metformin on the parenchyma cells directly in vitro (Fig. 4a; Supplementary Fig. S11), this result indicated that the mechanism by which metformin aggravates nephrotoxicity is likely non-cell autonomous. Our scRNA-seq profiling analyses showed that the percentage of renal neutrophils increased significantly in metformin-treated I/R mice (Fig. 4b, c). The intervention of metformin caused the response of neutrophils, and the number of circulating neutrophils increased significantly (Fig. 4d, e). Immunostaining of kidney sections for the neutrophil marker Ly6G confirmed that neutrophils were mobilized and expanded in the kidneys of I/R+Met mice (Fig. 4f). In contrast, a reduction in renal neutrophils was also observed in LKO+Met100 mice compared to Ctr+Met100 mice (Fig. 3s, t).

Next, to examine whether neutrophils played a role in metformin-increased I/R injury, we used the anti-Ly6G antibody to neutralize neutrophils. We found that the anti-Ly6G antibody virtually eliminated neutrophils in I/R+Met mice (Fig. 4g). Importantly, the anti-Ly6G antibody significantly improved the survival of I/R mice treated with 200 mg/kg metformin (Fig. 4h). In addition, we found that the anti-Ly6G antibody reduced renal hypertrophy (Fig. 4i), the kidney-to-body weight ratio (Fig. 4j) and biomarkers of renal function (Fig. 4k–n). Also, renal MDA level was decreased by intervention with anti-Ly6G (Fig. 4o). Further, renal histological such as fibrosis was improved in I/R mice treated with 100 mg/kg metformin by anti-Ly6G treatment (Fig. 4q). In particular, by Transmission Electron Microscopy (TEM), such treatment had an obvious protective effect on the morphology of renal tubular epithelial cells (Fig. 4q).

There is much evidence to show that neutrophils play a key role in aseptic injury^37^. The specific pathological form of neutrophils, NETosis, contributes to tissue damage when there is a high degree of neutrophils infiltrating^38^. As shown in Fig. 4p, q, neutrophil-specific myeloperoxidase (MPO) released during NETosis was significantly increased in the group of AKI mice treated with metformin, which was absent in the group treated with the neutralizing antibody. Similarly, another enzyme, neutrophil elastase, which is released specifically by neutrophils, increased significantly upon metformin treatment, and reversed by neutralizing antibody co-treatment (Fig. 4q). Furthermore, we found that treatment with the NETosis-specific inhibitors GSK484 and Cl-amidine could decrease the number of neutrophils in circulation (Fig. 4r, s), leading to the improvement of renal hypertrophy (Fig. 4t–z). Based on these evidence, we believe that neutrophils, with the participation of metformin, infiltrate into the site of kidney injury and undergo NETosis to promote metformin-aggravated nephrotoxicity. The anti-Ly6G antibody had similar renal protective effects in mice with rhabdomyolysis-induced AKI (Supplementary Fig. S12a–h).

### Interaction of renal NGAL with iron-bound metformin is required for accelerating AKI

**Fig. 5:**
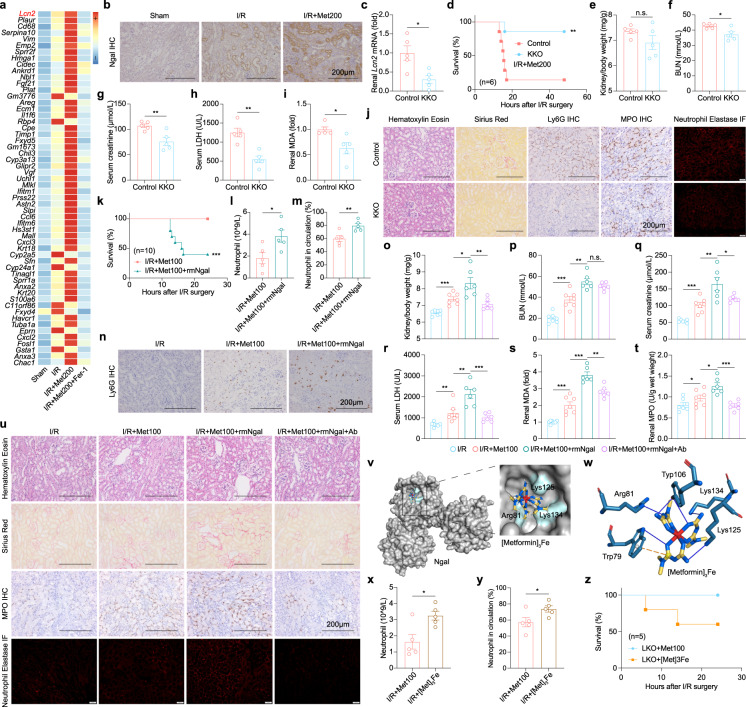
Kidney-derived NGAL exacerbates metformin nephrotoxicity and interacts with metformin–Fe. **a** The heatmap of cluster2 in Supplementary Fig. S10b and summary of *Lcn2* mRNA levels in sham group, I/R group, I/R+Met200 group, and I/R+Met200+Fer-1 group according to scRNA-seq data. **b** Representative NGAL IHC-stained kidney sections from the I/R, I/R+Met100 and I/R+Met100+rmNGAL groups. **c** Summary of renal *Lcn2* mRNA levels in *Lcn2* renal specific knockout mice (KKO) and control mice, expressed relative to control. **d** Kaplan–Meier survival curves of KKO and control mice in I/R+Met200 condition. **e** Summary of the kidney to body weight ratio measured in the I/R-induced KKO mice and control mice with metformin. **f**–**h** Summary of the serum BUN (**f**), creatinine (**g**), and LDH (**h**) measured in the KKO mice and control mice. **i** Summary of renal MDA levels measured in the KKO mice and control mice. **j** Representative H&E-stained, Sirius Red-stained, Ly6G IHC-stained, MPO IHC-stained, and Neutrophil Elastase IF-stained kidney sections from the KKO mice and control mice. **k** Kaplan–Meier survival curves of mice in I/R+Met100 and I/R+Met100+rmNGAL groups. **l** Summary of the neutrophil numbers in the circulation of the I/R+Met100 and I/R+Met100+rmNGAL mice. **m** Summary of the percentages of neutrophils in the circulation of the indicated mice. **n** Representative Ly6G IHC-stained kidney sections from the indicated mice. **o** Summary of the kidney to body weight ratio measured in I/R+Met100, I/R+Met100+rmNGAL, and I/R+Met100+rmNGAL+Ab mice. **p**–**r** Summary of serum BUN (**p**), creatinine (**q**), and LDH (**r**) measured in the indicated mice. **s** Summary of renal MDA levels measured in the indicated mice. **t** Summary of renal MPO levels measured in the indicated mice. **u** Representative H&E-stained, Sirius Red-stained, Ly6G IHC-stained, MPO IHC-stained, and Neutrophil Elastase IF-stained kidney sections from the indicated mice. **v** Structure of the NGAL protein complexed with metformin and Fe; the inset shows a zoomed in view of the electrostatic surface, with the key residues in NGAL indicated. **w** Forces between the indicated residues in the NGAL protein and the [Metformin]_3_Fe complex are shown. **x** Summary of the neutrophil numbers in the circulation of the mice with metformin or [Metformin]_3_Fe complex. **y** Summary of the percentages of neutrophils in the circulation of the indicated mice. **z** Kaplan–Meier survival curves of the LKO mice with metformin or [Metformin]_3_Fe complex (*n* = 5 mice per group). *P < 0.05, **P < 0.01, ***P < 0.001 and n.s., not significant (one-way ANOVA). Survival curve comparison was analyzed by a log-rank Mantel-Cox test. In this and subsequent figures, summary data are represented as the mean ± SEM. See also Supplementary Figs. S13–S17.

It is still unclear how neutrophils are attracted to the kidney during metformin-aggravated AKI. Based on our single-cell transcriptomic data (Supplementary Fig. S13a–q), the *Lcn2* gene (encoding NGAL protein), was identified as the most significantly upregulated gene in several kidney parenchymal cells (Fig. 5a) or kidneys (Fig. 5b). We therefore tested whether knocking out endogenous *Lcn2* in the kidney (KKO mice) (Fig. 5c) could rescue the progression of AKI in I/R+Met mice. Indeed, In KKO mice, metformin-induced death was significantly prevented (Fig. 5d). Further, by serum biochemistry, KKO mice showed an alleviation of kidney damage with metformin (Fig. 5e–h), including reduced ferroptosis as measured by lower renal MDA (Fig. 5i) and less pathology, neutrophil infiltration, and NETosis (Fig. 5j).

By a gain of function approach, we found that treating mice with recombinant mouse NGAL (rmNGAL) promoted metformin-aggravated nephrotoxicity (Fig. 5k). Consistent with the I/R model, we also found that rmNGAL reduced the survival of metformin-treated mice subjected to rhabdomyolysis-induced AKI (Supplementary Fig. S14a, b).

It is worth emphasizing that the intervention of rmNGAL caused the aggregation of neutrophils in circulation (Fig. 5l, m) and the kidneys (Fig. 5n). However, use of the anti-Ly6G neutralizing antibody of neutrophils prevented the exacerbated kidney damage induced by rmNGAL (Fig. 5o–r), as well as resulting in lower renal MDA (Fig. 5s), MPO (Fig. 5t, u) and neutrophil elastase levels (Fig. 5u). From the pathological results of the survivors, we observed that rmNGAL aggravated the morphological changes of renal parenchymal cells (Fig. 5u). When we used NGAL protein to directly treat ferroptotic parenchymal cells there were no obvious effects (Supplementary Fig. S15a–c), suggesting NGAL protein released by damaged renal parenchymal cells does not act in an autocrine manner. Together, these results suggest that NGAL has a certain role, even if it is indirect, in the infiltration and accumulation of neutrophils into the injured kidney that in turn facilitate NETosis.

It is important to note that although NGAL was upregulated in the I/R group, neutrophils accumulated only in the I/R+Met group. As a secreted protein with anti-infective properties, NGAL not only binds to siderophore-Fe, but also interacts with small organic molecules such as catechol^39^. Interestingly, metformin molecules were fully reacted with ferric chloride, obtaining a yellow-brown precipitate in the ethanol as the organic-reaction environment (Supplementary Fig. S16a). Using chemical element analysis, we found that three metformin molecular form a complex with one Fe ion (Supplementary Fig. S16b–d). As Solier and colleagues recently reported^40^, the modified molecules of metformin could bind to copper ions and maintain the immune function of macrophages. Together, these findings support the important regulatory functions of the complex formed by metformin-bound metal ions, such as copper and iron in vivo. Based on previous molecular docking data^39^, we used the structure simulation to construct the NGAL bound to the metformin–Fe complex via the residues including Arg81, Lys125, and Lys134 (Fig. 5v, w; Supplementary Fig. S16e). In addition, the NGAL–metformin–Fe complex was more stable than the catechol–Fe complex (Supplementary Fig. S16f, g), because more energy was released upon formation of the NGAL–metformin–Fe complex (Supplementary Fig. S16h). To verify that the NGAL–metformin–Fe complex has a physiological function, we injected [Metformin]_3_Fe complex into I/R mice and found that it had a significant effect on the mobilization of neutrophils (Fig. 5x, y).

Our results above showed that low-iron status mice (LKO) could protect against metformin-aggravated nephrotoxicity, while effectively reducing the renal aggregation of neutrophils. So, we tested if the intervention of the NGAL–metformin–Fe complex could reverse the phenotype of LKO mice. Based on serum results, we found the complex could effectively reverse the protective effect of LKO on metformin-aggravated nephrotoxicity (Supplementary Fig. S17a–f) and cause a certain degree of death in LKO mice (Fig. 5z).

### CXCR4 mobilizes neutrophils to the site of renal damage by recognizing NGAL during metformin-aggravated AKI

**Fig. 6:**
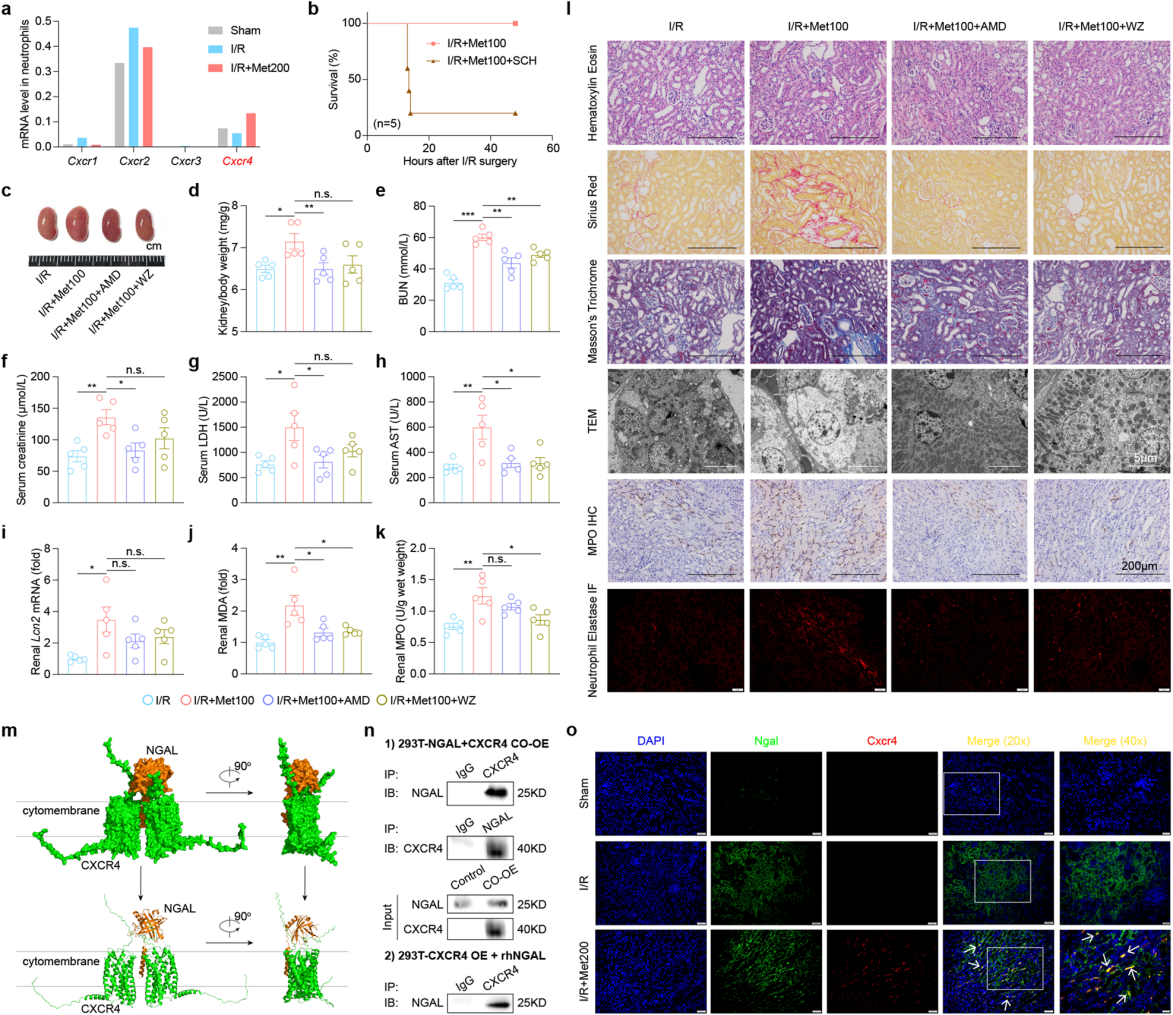
Mobilization of neutrophils to the site of kidney damage via CXCR4 is mediated by NGAL–metformin–Fe. **a** The *Cxcr* family mRNAs were measured in neutrophils obtained from scRNA-seq data. **b** Kaplan–Meier survival curves of the I/R+Met100 group and I/R+Met100+SCH group. **c** Representative image of kidneys removed from a mouse following I/R, a mouse in I/R+Met100, a mouse in I/R+Met100+AMD (5 mg/kg), and a mouse in I/R+Met100+WZ (4 mg/kg) group. **d** Summary of the kidney to body weight ratio measured in the indicated mice. **e**–**h** Summary of serum BUN (**e**), creatinine (**f**), LDH (**g**), and AST (**h**) measured in the indicated mice. **i** Summary of the *Lcn2* mRNA levels measured in the indicated mice, expressed relative to I/R. **j** Summary of the renal MDA levels measured in the indicated mice. **k** Summary of the renal MPO levels measured in the indicated mice. **l** Representative H&E-stained, Sirius Red-stained, Masson’s Trichrome-stained, MPO IHC-stained, Neutrophil Elastase IF-stained, and electron micrographs showing kidney sections from the indicated mice. **m** Cryo-EM density of CXCR4 (green) and NGAL (orange). NGAL binds to an extracellular facing cavity in CXCR4. **n** CXCR4 and NGAL were either co-expressed (Top panel) or only expressing CXCR4 and then co-cultured with recombinant human NGAL (rhNGAL) (Bottom panel) in HEK293T cells; 48 h after transfection, the cell lysates were immunoprecipitated using protein A/G beads to pull-down CXCR4 or NGAL (IP), respectively, followed by immunoblotting (IB) with the other antibody. Immunoglobulin G was used as a negative control, and the input was used a positive control. **o** Green asterisk labels the NGAL, red asterisk labels the CXCR4, then analyzed using immunofluorescence. *P < 0.05, **P < 0.01, ***P < 0.001 and n.s., not significant (One-way ANOVA). Survival curve comparison was analyzed by a log-rank Mantel-Cox test. In this and subsequent figures, summary data are represented as the mean ± SEM. See also Supplementary Figs. S18 and S19.

The chemokine receptor CXCR1 through CXCR4, which are expressed on the surface of neutrophils, have been shown to play roles in tissue damage^41,42^. Among the four CXCR-encoding genes, we found that only the change of *Cxcr4* was consistent with the observed murine phenotype (Fig. 6a), which is important for the trafficking of neutrophils from the bone marrow^41^. We also ruled out the possibility that CXCR1 and/or CXCR2 play a role, as specific CXCR1/2 antagonist SCH527123 caused significant lethality when given to I/R+Met mice (Fig. 6b). In contrast, the CXCR4-specific inhibitors AMD3100 and WZ811 significantly reduced the signs associated with nephrotoxicity in I/R+Met mice, including renal hypertrophy (Fig. 6c, d); increased renal biomarkers such as serum BUN (Fig. 6e), creatinine (Fig. 6f), LDH (Fig. 6g), AST (Fig. 6h) and *Lcn2* mRNA levels (Fig. 6i); elevated renal MDA, a marker of ferroptosis‒induced neutrophil mobilization and expansion of neutrophils (Fig. 6j); and renal fibrosis (Fig. 6l). Additionally, the subcellular structure, such as mitochondria (Fig. 6l), of renal tubular epithelial cells was protected by CXCR4 inhibitors. When we inhibited *Cxcr4*, we found a reduced accumulation of neutrophils, and subsequently NETosis was also blocked (Fig. 6k, l).

The migration of neutrophils requires ligand signaling from the CXCL family. Thus, we used the CXCL family inhibitor UNBS5162 to explore the role of such signaling on neutrophil accumulation during metformin-induced nephrotoxicity. We found that inhibition of CXCL did not prevent metformin-aggravated nephrotoxicity (Supplementary Fig. S18a–f). As the neutrophil response is only present during metformin exposure, the continuous rise of NGAL at the same time may act as a recognized substance. Surprisingly, using protein molecular docking analysis, we found that NGAL could bind with CXCR4 (Fig. 6m). We confirmed this binding by coimmunoprecipitation (co-IP) (Fig. 6n) and the metformin-Fe complex could increase the degree of binding (Supplementary Fig. S19a, b). We also performed immunofluorescence-mediated co-localization of CXCR4 and NGAL in kidney sections and found that NGAL staining overlaps with CXCR4 (Fig. 6o). These results suggest that NGAL, especially when complex with metformin–Fe, acts as a ligand for CXCR4 to promote neutrophil accumulation to sites of renal damage.

## Discussion

In this study, we originally aimed to identify molecular targets to treat CKD, one of the crucial morbidities of human aging that limits lifespan. As AKI is often a key risk factor for CKD, we explored the effects of various previously identified longevity drugs on the progression of AKI in mice. Unexpectedly, we found that compared with other longevity drugs, metformin not only had no therapeutic effect on two AKI mouse models, but it also in fact aggravated the degree of injury. Interestingly, reports have suggested that metformin can have clinical benefits in kidney diseases^22,43^. Conversely, a recent review found that high doses of metformin can have detrimental effects on patients with diabetic nephropathy^44^. Consistently, several cohort studies and our meta-analysis (Supplementary Fig. S4) suggested that metformin might exacerbate kidney injury^45^. Further, in a case report a patient with type 2 diabetes on metformin developed significant kidney aggravation and systemic organ acidosis^46^. But a growing number of non-diabetic people are taking daily doses of metformin to increase their lifespan and to improve weight and appetite control, but without monitoring their kidney function. So subtle toxicity cannot be ruled out. Undoubtedly, this is an issue that deserves attention and discussion.

Based on our functional results obtained using two separate mouse models of AKI, we conclude that for the three dose of metformin we tested there was exacerbation of renal toxicity in the context of AKI. Several forms of cell death have long been implicated in the pathophysiology of AKI; however, using pharmacological inhibitors of ferroptosis, necroptosis, and apoptosis in our AKI models, we found that metformin-aggravated nephrotoxicity is mediated primarily by ferroptosis of renal parenchymal cells.

To understand the role of specific renal cell types and transcriptional changes at the single-cell level in I/R+Met mice, we performed scRNA-seq on kidney cells from indicated mice. In addition to the multiple cell death of proximal tubular cells, which fits with prior studies on AKI (ferroptosis^31^, necroptosis^47^, apoptosis^48^, and pyroptosis^49^), we also found that massive expansion of neutrophils and the induction of NETosis, thus contributing in important ways to metformin-aggravated nephrotoxicity. Importantly, depleting neutrophils significantly improved survival in I/R+Met mice, indicating that neutrophils play a causal role in driving the progression of metformin-aggravated AKI.

Mechanistically, we found that the protein NGAL forms a complex with iron-bound metformin, thus driving neutrophils infiltration into the kidney, which eventually results in NETosis. We also found that CXCR4-expressing neutrophils are mobilized by the NGAL-metformin-Fe complex released from ferroptotic renal parenchymal cells during AKI. Based on our results, we conclude that neutrophils serve as an important trigger driving metformin-aggravated nephrotoxicity, paving the way to better understanding the pathophysiological processes by which metformin causes nephrotoxicity.

In conclusion, our data provide compelling evidence that metformin can induce nephrotoxicity, particularly in the context of AKI, which is important as episodes of AKI are known to strikingly increase the risk of developing CKD. In addition, our results provide several potential therapeutic targets for the prevention and/or treatment of metformin-induced nephrotoxicity, including not only diabetic nephropathy. From a clinical perspective, our findings suggest that systemic NGAL levels and iron levels should be monitored in patients receiving metformin, and these potentially nephrotoxic effects should be highlighted in the metformin label, in addition to monitoring the patient’s renal function. Likewise, if metformin is going to be used as an anti-aging medication, effects on renal function need to be highly considered.

## Materials and methods

### Animal models

Male and female C57BL/6J mice were purchased from GemPharmatech. Where indicated, starting at 3 weeks of age, the mice were fed a standard AIN-76A diet containing 50 mg iron/kg (Research Diets, Inc., New Brunswick, NJ), a low-iron diet (LID; 0.9 mg iron/kg) and a high-iron diet (HID; 8.3 g carbonyl iron/kg) for 9 weeks. LKO mice (*Tmprss6*^*Alb/Alb*^) were generated by crossing *Tmprss6*^*flox/flox*^ and *Alb-Cre* transgenic mice. Kidney-specific *Lcn2* knockout mice (*Lcn2*^*Six2/Six2*^) were generated by crossing *Lcn2*^*flox/flox*^ and *Six2-Cre* transgenic mice. All mice were housed under a 12-h light-dark diurnal cycle at 23–25 °C with 40%–60% relative humidity. Food and water were available ad libitum. All experiments involving animals were performed in accordance with the National Institutes of Health’s Guide for the Care and Use of Laboratory Animals and were approved by the Animal Care and Use Committee of Zhejiang University.

### Animal treatments and related measurements

#### Renal I/R injury model

Mice were anesthetized with isoflurane (RWD Life Science) and placed in a supine position on a heating pad to maintain a core temperature of 37 °C. The kidneys were exposed via flank incisions, and both renal pedicles were clamped for 45 min using microaneurysm clamps (RWD Life Science) to induce transient ischemia. After the clamps were released (for reperfusion), the kidneys were visually inspected for a color change, and restoration of blood flow was confirmed before closing the incisions with surgical staples. During ischemia, the surgical incision and kidneys were covered with sterile saline-soaked tissues to minimize evaporative loss. Sham-operated mice underwent the same procedure, except the clamps were not closed. The mice were sacrificed either 13 h or 24 h after I/R, and the kidneys were harvested for mRNA analysis or embedded in paraffin for staining. Blood was also collected via cardiac puncture, and serum AST (aspartate aminotransferase), BUN (blood urea nitrogen), creatinine, and LDH (lactate dehydrogenase) levels were measured using a biochemistry analyzer (Sysmex) at the Center for Drug Safety Evaluation and Research, Zhejiang University.

#### Rhabdomyolysis

Rhabdomyolysis was induced using the glycerol model in 8–10 weeks-old mice as described previously^50^. In briefly, the mice were deprived of water for 16 h, after which the mice were anesthetized with isoflurane and given an injection of 50% glycerol in water (or saline as a control) at a volume of 7.5 mL/kg body weight (half the volume delivered into each anterior thigh muscle). The mice were sacrificed 24 h after injection, and the kidneys were harvested for mRNA analysis or embedded in paraffin for staining. Blood was collected via cardiac puncture, and serum AST, BUN, creatinine, and LDH were measured as described above.

#### In vivo drug treatment

Metformin HCl (T0740, TargetMol, 50 mM o.a., 50 mg/kg i.p., 100 mg/kg i.p. or 200 mg/kg i.p.), Fe[Metformin]_3_ (33.3 mg/kg) were administered intraperitoneally at the indicated times; or (50 mM metformin) was supplied either in drinking water at desired concentrations or in standard diet at 0.1% (w/w) for 1 week. Nicotinamide (50 mg/kg, TargetMol), resveratrol (100 mg/kg, TargetMol), rapamycin (10 mg/kg, TargetMol), dasatinib (5 mg/kg, TargetMol), and quercetin (50 mg/kg, TargetMol) were administered intraperitoneally at the indicated times before I/R. Fer-1 (S7243, Selleck, 1 mg/kg), Lip-1 (S7699, Selleck, 1 mg/kg), Nec-1 (S8037, Selleck, 1 mg/kg), Nec-1s (S8641, Selleck, 1 mg/kg), Emricasan (S7775, Selleck, 2.5 mg/kg) were administered intraperitoneally at the indicated times before I/R^33^. GSK484 (S7803, Selleck, 20 mg/kg)^51^ and Cl-amidine (S8141, Selleck, 75 mg/kg)^52,53^ were administered intraperitoneally at the indicated times before I/R. SCH527123 (S8506, Selleck, 50 mg/kg)^54^, AMD3100 HCl (S3013, Selleck, 5 mg/kg)^55^, WZ811 (S2912, Selleck, 4 mg/kg)^56^, UNBS5162 (S8869, Selleck, 50 mg/kg)^57^ were administered intraperitoneally at the indicated times before I/R. rmNGAL (CM17, Novoprotein Scientific Inc., 500 µg/kg) was injected intravenously 2 h prior to I/R. The interval between each drug injection and metformin injection was at least 30 min. Neutrophils were depleted by an intraperitoneal injection of 200 μg of anti-Ly6G (1A8, Bio X Cell) antibody 24 h prior to I/R^37^.

### scRNA-seq

#### Preparation of single-cell suspensions

Mouse kidney tissues were dissociated using the mouse Kidney Dissociation Kit (130-110-207, Miltenyi Biotec) in accordance with the manufacturer’s instructions. Cell viability was confirmed by Trypan Blue exclusion. The cell suspension was loaded onto a Chromium single-cell controller (10X Genomics) to generate single-cell gel beads in the emulsion in accordance with the manufacturer’s instructions.

#### scRNA-seq processing and analysis

The Cell Ranger software was download from the 10x Genomics website. and the Mouse Reference Dataset version mm10 was used. Alignment, filtering, barcode counting, and UMI (unique molecular identifier) counting were performed using the cellranger count module to generate a feature-barcode matrix, to determine clusters, and finally to obtain a total number of 1,000,336 estimate cells from 4 groups (8 individuals) which were acquired after the AGGR procedure. Dimensionality reduction was performed using principal component analysis (PCA) and *t*-SNE, where the first ten principal components were used to generate clusters using the K-means algorithm and the graph-based algorithm, respectively. Cell types within the kidney cell population were identified manually using combinations of marker gene derived from the literatures and aided with the Seurat package using previously published datasets as a reference to assign the cell type automatically^27^. Significant genes among clusters or between groups were identified using the software Loupe Brower from 10X Genomics with the “Locally Distinguish” option.

### Flow cytometry

Cells were isolated from the spleen 24 h after surgery using the Mouse Kidney Dissociation Kit (Miltenyi Biotec) in accordance with the manufacturer’s instructions; cells were also isolated from the bone marrow by rinsing with sterile PBS. The proportion of neutrophils was then analyzed using flow cytometry (ACEA NovoCyte) with the anti-Ly6G antibody (Biolegend), anti-Gr-1 antibody (Biolegend), and Zombie (Biolegend) for 30 min at 4 °C. All flow cytometry data were analyzed using FlowJo software (Tree Star).

### RNA isolation, and RT-PCR

Total RNA was isolated from tissues or cells using Trizol (Pufei), and RNA concentration and purity were measured using a spectrophotometer. RNA was reverse-transcribed using the Strand cDNA Synthesis SuperMix (Yeasen) in accordance with the manufacturer’s instructions, and quantitative PCR was performed using a CFX96 Real-Time System (Bio-Rad) with SYBR Green SuperMix (Vazyme) in accordance with the manufacturer’s instructions. The recommended thermal protocol consisted of an initial denaturation step at 95 °C for 3 min, followed by 40 cycles of denaturation at 95 °C for 15 s, annealing at 60 °C for 20 s, and extension at 72 °C for 30 s. The fold difference in gene expression was calculated using the 2^-△△Ct^ method and is presented relative to *ACTB* (*β-Actin*) mRNA. All reactions were performed in triplicate, and specificity was monitored using melting curve analysis.

### Histology

The right kidney was removed, fixed for at least 24 h in 4% paraformaldehyde (pH 7.4), embedded in paraffin, and serially sectioned at 5-µm thickness. The sections were then stained with H&E, Sirius Red, Masson’s Trichrome for routine histological examination using a light microscope. Representative images were selected based on the value closest to the mean value of each group.

### IHC

The anti-Ly6G antibody (1:200, Cell Signaling), anti-Lipocalin-2/NGAL (1:1000, abcam) were used for IHC. Images were taken using Nikon ECLIPSE Ni-U. Image J software (NIH) was used for process and quantify Ly6G-positive areas in the images.

### co-IP

co-IP experiments were performed as previously described^58^. NGAL was cloned into the pEZT vector with a strep tag, and CXCR4 was cloned into the pEnCMV-3× Flag vector. The constructs were co-expressed in HEK293T cells; 48 h after transfection, the cell lysates were prepared and immunoprecipitated using protein A/G beads conjugated with CXCR4 or NGAL antibody to pull-down CXCR4 or NGAL, respectively, followed by immunoblotting with the other antibody. Immunoglobulin G was used as a negative control, and the input was used a positive control. The following primary antibodies were used for the co-IP and western blot analysis: anti-CXCR4 (1:1000, abcam), anti-NGAL (1:1000, abcam).

### Measurements of iron parameters

Serum iron was measured as described^59,60^. Tissue non-heme iron levels were measured using the chromogen method^61^. In brief, the tissues were weighed and digested in 10% trichloroacetic acid in 3 M HCl for 48 h at 65–70 °C. Equal volumes of sample and iron standard (500 μg/dL) were incubated for 10 min at room temperature in 200 μL BAT buffer (0.2% thioglycolic acid and 0.02% disodium-4,7-diphenyl-1,10-phenanthroline disulfonate in 50% saturated NaAc solution). The samples were read at 535 nm, and values were calculated using a standard curve. The results are presented in micrograms of iron per gram of wet tissue.

### Compound synthesis

Eqimolar amounts of metformin hydrochloride (1115-70-4, Collaber Science & Technology) and sodium hydroxide (NaOH, 1310-73-2) were dissolved by stirring in ethanol at room temperature for 24 h; ultra-fine filter paper was then used to filter out the sodium chloride produced by the reaction, and rotary steaming in a 50 °C water bath was performed to obtain pure metformin. The same molar amount of ferric chloride (157740, Sigma) was then stirred with the pure metformin in ethanol at room temperature for at least 48 h. The solid phase and liquid phase were then separated by suction filtration, and the solid phase was dried to a constant weight and then subjected to elemental analysis using a Vario MICRO cube to obtain the ratio of metformin and iron in the resulting complex.

### Molecular docking and ligand–protein interaction analysis

The receptor protein NGAL used for molecular docking was constructed based on the NGAL complex (PDB ID: 1L6M, https://www.rcsb.org/) without ligands. KingDraw (https://www.kingdraw.com) was used for creating the docked ligands, including iron-bound metformin and iron-bound dicatechol.

The chain A of the receptor protein NGAL was used as the docking target chain. The active sites were identified by the docking box built around the deleted proligand. Molecular docking was processed by Ledock (http://www.lephar.com/) and the ligand–protein interaction analysis was carried out by PLIP (https://plip-tool.biotec.tu-dresden.de/). Open source PyMOL (https://github.com/schrodinger/pymol-open-source) and Openbabel (openbabel.org) were used for visualization and format conversion, respectively.

### Molecular modeling and protein–protein interaction analysis

The protein sequences of CXCR4 (P61073) and NGAL (P80188) were acquired from Uniprot (https://www.uniprot.org/). The modeling of CXCR4-NGAL protein interaction was predicted by AlphaFold2 through COSMIC2 platform (https://cosmic-cryoem.org/)^62^, followed by analyzing the reciprocal residues using LigPlot+ (https://www.ebi.ac.uk/thornton-srv/software/LIGPLOT/) and PyMol for visualization.

### Meta-analysis

#### Search strategy

The databases PubMed, Embase, and Web of Science were searched for all articles published through Feb 20th, 2023, with no language restrictions, using the following keywords: “metformin kidney injury” OR “metformin kidney disease” OR “metformin renal impairment” OR “metformin renal disease”.

#### Study selection

The systematic review and meta-analysis were conducted according to PRISMA guidelines. Studies that satisfied the following three criteria were included in our meta-analysis: (1) the study was a clinical observation in humans; (2) the study included metformin used patient information; and (3) the study included information regarding AKI, CKD, nephropathy, renal impairment or other kidney diseases, eGFR level, and creatinine level. In addition, we excluded studies that were published as a narrative review, comment, opinion piece, methodological reports, editorials, letters, or conference abstracts.

#### Data extraction

Data were extracted using a standardized data collection form. Detailed information was extracted from each included article, including the first author, publication date, study design, patients’ age, sample size, type of kidney diseases, and renal indicators.

### Statistical analysis

Statistical analyses were performed using GraphPad Prism version 8.0, and all summary data were presented as the mean ± SEM for each independent experiment. Groups were compared using the Student’s *t*-test (for comparing two groups) or one-way ANOVA (for multi-groups comparisons). Significance in survival curves was calculated using the log-rank (Mantel-Cox) test. The Differences were considered statistically significant at *P < 0.05, **P < 0.01, ***P < 0.001.

Meta-analyses were conducted in order to evaluate the putative association between metformin use and various kidney diseases^63^. The pooled results for use in the forest plots were analyzed using a random-effects model. All statistical analyses were performed using Stata statistical software version 12 (StataCorp).

### Supplementary information

Supplementary, Figures and Tables

## Data Availability

All raw sequencing data and processed data generated in this work are deposited in the GEO database (GSE240844). Further information on materials, datasets, and protocols should be directed to fwang@zju.edu.cn.
