# Author Correction: Metformin potentiates nephrotoxicity by promoting NETosis in response to renal ferroptosis

**DOI:** 10.1038/s41421-023-00630-3

**Published:** 2024-01-08

**Authors:** Zhaoxian Cai, Xiaotian Wu, Zijun Song, Shumin Sun, Yunxing Su, Tianyi Wang, Xihao Cheng, Yingying Yu, Chao Yu, En Chen, Wenteng Chen, Yongping Yu, Andreas Linkermann, Junxia Min, Fudi Wang

**Affiliations:** 1 The Second Affiliated Hospital, The First Affiliated Hospital, School of Public Health, Institute of Translational Medicine, Cancer Center, Zhejiang University School of Medicine, Hangzhou, Zhejiang China; 2 The First Affiliated Hospital, Basic Medical Sciences, School of Public Health, Hengyang Medical School, University of South China, Hengyang, Hunan China; 3 College of Pharmaceutical Science, Zhejiang University, Hangzhou, Zhejiang China; 4 Division of Nephrology, Department of Internal Medicine III, University Hospital Carl Gustav Carus at the Technische Universität Dresden, Dresden, Germany; 5 Division of Nephrology, Department of Medicine, Albert Einstein College of Medicine, Bronx, NY USA

Correction to: *Cell Discovery* (2023) 9:104

10.1038/s41421-023-00595-3, published online 17 October 2023

In the original publication of this article, we inadvertently misplaced an incorrect image for representative H&E-stained kidney sections from I/R+Met100 mice group in Fig. 2i. The correct Fig. 2i is displayed as below. This correction does not affect the results or the conclusion of this work. We are sorry for any inconvenience that might cause.
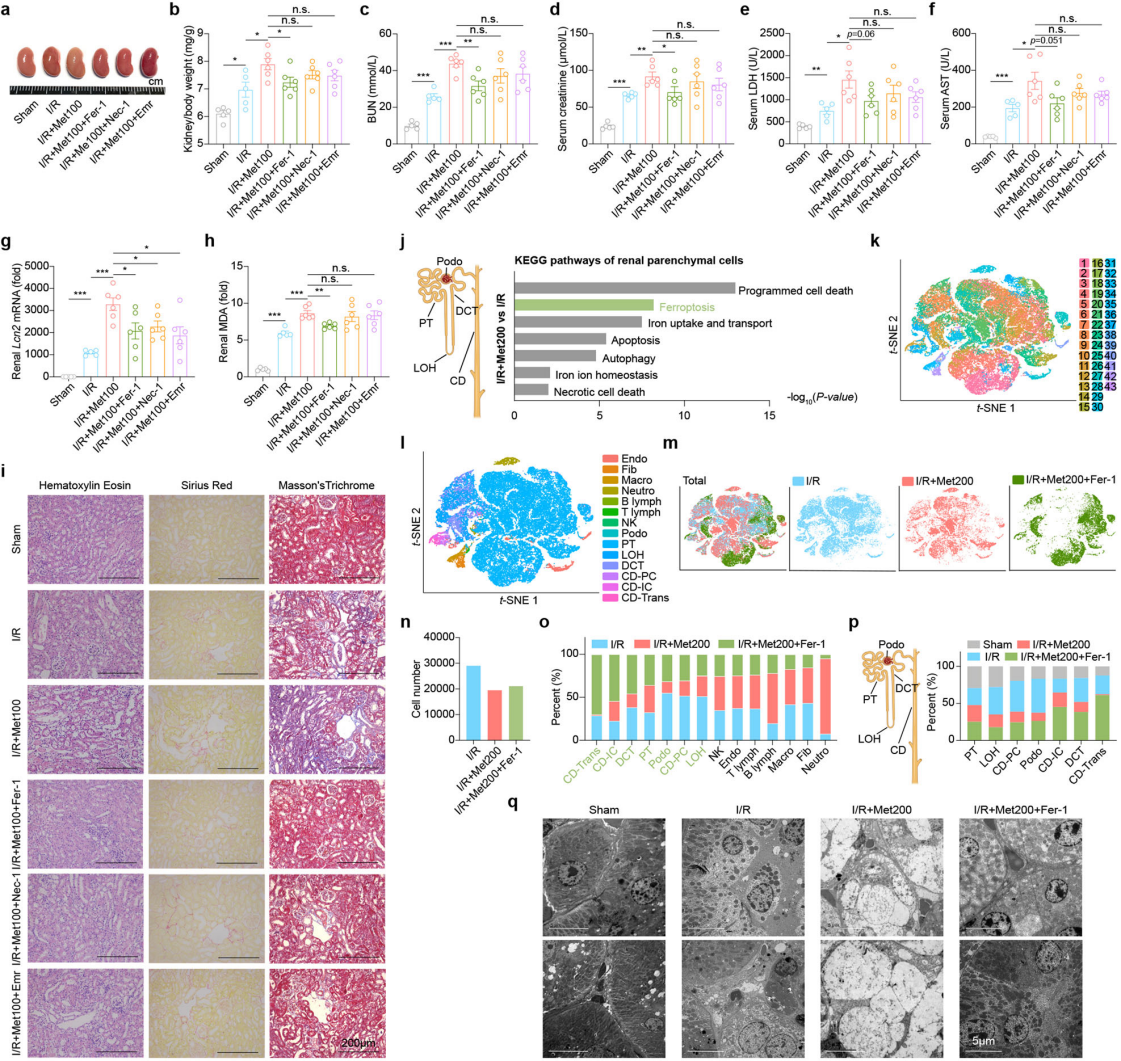


**Fig. 2 a** Representative image of kidneys removed from mice in the indicated groups. Where indicated, the mice received metformin (100 mg/kg), Fer-1 (1 mg/kg), Nec-1 (1 mg/kg), or Emr (2.5 mg/kg). **b** Summary of the kidney to body weight ratio measured in the indicated mice. **c**–**f** Summary of serum BUN (**c**), creatinine (**d**), LDH (**e**), and AST (**f**) measured in the indicated mice. **g** Summary of renal Lcn2 mRNA levels measured in the indicated mice. **h** Summary of renal MDA levels measured in the indicated mice. **i** Representative H&E-stained, Sirius Red-stained, and Masson’s Trichrome-stained kidney sections from the indicated mice. **j** Summary of significantly differential pathways in the indicated parenchymal cell types of I/R + Met200 vs I/R. **k** t-SNE reveals clustering of 69,608 renal cells obtained from mice in the I/R group (*n* = 2), I/R + Met200 group (*n* = 2), and I/R + Met200 + Fer-1 group (*n* = 2). **l** *t*-SNE reveals 14 distinct cell types in the kidney. **m** Annotation of renal cells in I/R, I/R+Met200 and I/R+Met200+Fer-1 groups. **n** Summary of the number of cells included in the scRNA-seq analysis for the indicated groups (*n* = 2 mice per group), expressed relative to the Sham. **o** Summary of the percentages of the indicated cell types in the indicated groups. **p** Summary of the percentage of the indicated renal parenchymal cell types in four groups shown at the left. **q** Electron micrographs showing the cellular morphology of renal tubular epithelial cells in the indicated groups. *P < 0.05, **P < 0.01, ***P < 0.001, and n.s., not significant (One-way ANOVA). See also Supplementary Figs. S5–S8.

