# Supplementary material for: Metformin potentiates nephrotoxicity by promoting NETosis in response to renal ferroptosis
Source: Cell Discov. 2023 Oct 17;9:104. doi: 10.1038/s41421-023-00595-3 (PMC10582023; doi:10.1038/s41421-023-00595-3)
Supplement: Supplementary file 1 — Supplementary, Figures and Tables [file 41421_2023_595_MOESM1_ESM.pdf]

Supplementary Information

Metformin Potentiates Nephrotoxicity by Promoting NETosis in Response to Renal Ferroptosis

Zhaoxian Cai[#], Xiaotian Wu[#], Zijun Song[#], Shumin Sun, Yunxing Su, Tianyi Wang, Xihao Cheng, Yingying Yu, Chao Yu, En Chen, Wenteng Chen, Yongping Yu, Andreas Linkermann, Junxia Min[*], Fudi Wang[*]

This file includes:

- Supplementary Materials
- Supplementary Figures S1-19
- Supplementary Tables S1, 2

Supplementary Materials

Reagent or resource	Source	Identifier
Chemicals, Recombinant Proteins		
Metformin hydrochloride	TargetMol	CAT# T0740
Nicotinamide	TargetMol	CAT# T0934
Resveratrol	TargetMol	CAT# T1558
Rapamycin	TargetMol	CAT# T1537
Dasatinib	TargetMol	CAT# T1448
Quercetin	TargetMol	CAT# T2174
Ferrostatin-1 (Fer-1)	Selleck	CAT# S7243
Necrostatin-1 (Nec-1)	Selleck	CAT# S8037
Emricasan (IDN-6556)	Selleck	CAT# S7775
GSK484 hydrochloride	Selleck	CAT#S7803
Cl-amidine	Selleck	CAT#S8141
SCH527123 (Navarixin)	Selleck	CAT# S8506
AMD3100 (Plerixafor) HCl	Selleck	CAT# S8030
WZ811	Selleck	CAT# S2912
UNBS5162	Selleck	CAT# S8869
Recombinant mouse NGAL (C-6His)	Novoprotein	CAT# CM17; Lot 0332142HF09
$FeCl_3$	Sigma-Aldrich	CAT# 157740; CAS [7705-08-0]
Glycerol	Sinopharm Chemical Reagent	CAT# 10010618; CAS [56-81-5]
**Antibodies**		
*InVivo*MAb anti-Ly6G	Bio X Cell	CAT# BE0075-1
PE anti-mouse Ly6G antibody	Biolegend	CAT# 127607
Pacific Blue anti-mouse Ly6G/Ly6C (Gr-1) antibody	Biolegend	CAT# 108429
Zombie Green Fixable Viability Kit	Biolegend	CAT# 423111

Rabbit mAb anti-Ly6G	Cell Signaling	CAT# 87048S
Anti-CXCR4 antibody	abcam	CAT# ab181020
Anti-Lipocalin-2/NGAL antibody	abcam	CAT# ab125075
Anti-Lipocalin-2/NGAL antibody	abcam	CAT# ab216462
Anti-Lipocalin-2/NGAL antibody	abcam	CAT# ab70287
Anti-Myeloperoxidase antibody	abcam	CAT# ab208670
Anti-Neutrophil Elastase antibody	abcam	CAT# ab68672
**Experimental models: Organisms/strains**		
Mouse: C57BL/6JGpt	GemPharmatech	CAT# N000013
Mouse: C57BL/6-Tmprss6em1(flox)Smoc	Shanghai Model Organisms	CAT# NM-CKO-2116243
Mouse: C57BL/6-Lcn2tm1 (flox)Smoc	Shanghai Model Organisms	CAT# NM-CKO-00134
Mouse: C57BL/6-Six2 (Cre)Amc/J	The Jackson Laboratory	CAT# 009606
Mouse: C57BL/6-Albem1(IRES-iCre-WPRE-pA)Smoc	Shanghai Model Organisms	CAT# NM-KI-200075
**Software and algorithms**		
FlowJo v.10.4.1	FlowJo	https://www.flowjo.com/
GraphPad Prism v8	GraphPad	https://www.graphpad.com/scientific-software/prism/
ImageJ	NA	https://imagej.net/Fiji/Downloads
Loupe Browser	10X Genomics	https://www.10xgenomics.com/products/loupe-browser
**Other**		
Single cell RNA Sequencing	CapitalBio Technology	https://www.capitalbiotech.com/

Supplementary Fig. S1

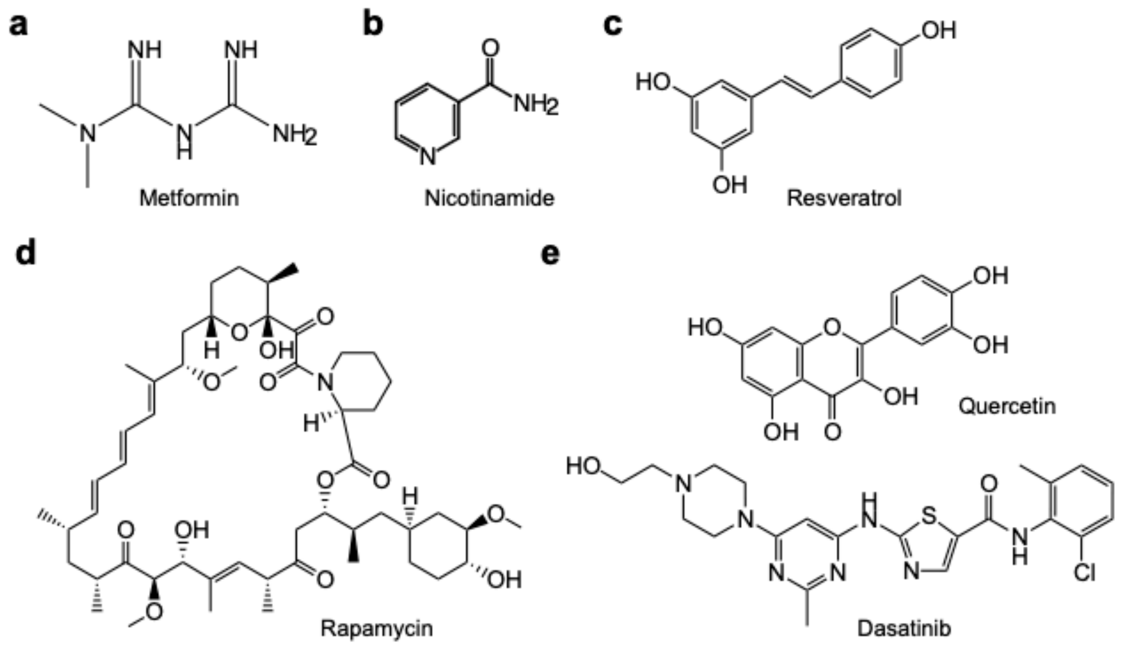

Supplementary Fig. S1 I Two-dimensional chemical structures of the longevity drugs in Figure 1.

Supplementary Fig. S2

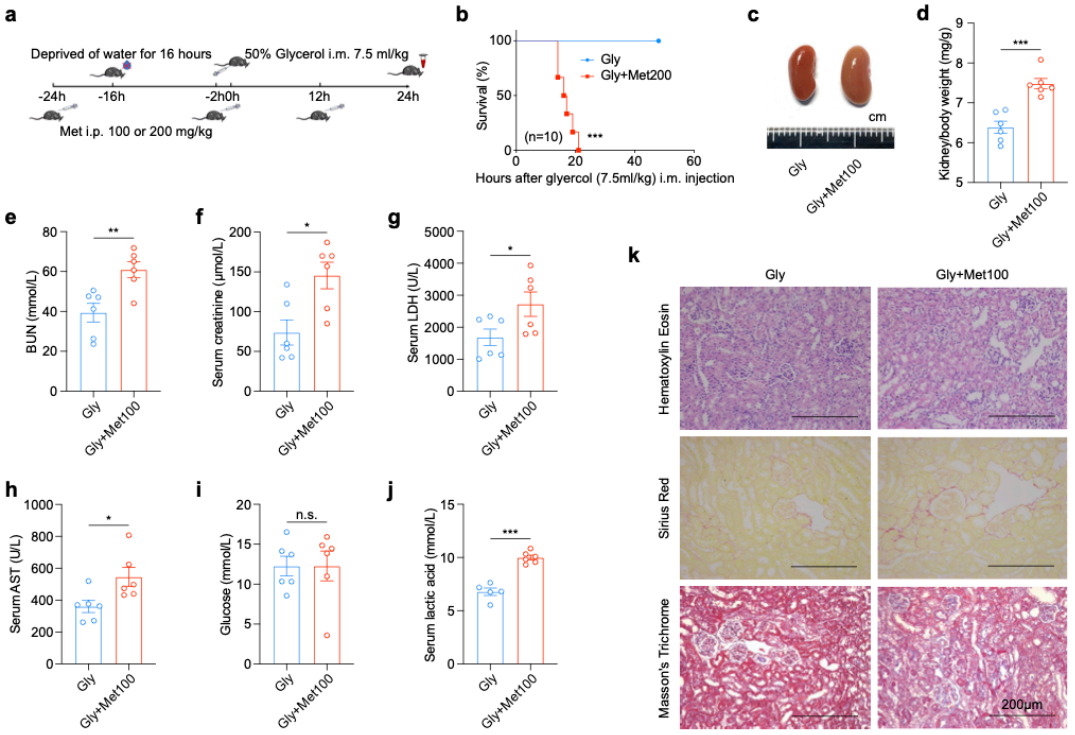

Supplementary Fig. S2 | Metformin aggregated rhabdomyolysis-induced AKI in mice. a. Overview of the experimental procedure used to induce rhabdomyolysis in mice; where indicated, i.p. injections of metformin (100 mg/kg or 200 mg/kg) were administered. **b**. Kaplan-Meier survival curves of the indicated groups (n=10 mice per group). **c**. Representative image of kidneys removed from a mouse following Gly (Glycerol 7.5 ml/kg i.m.) and Gly+Met100 (100 mg/kg) groups. **d**. Summary of the kidney to body weight ratio measured in the indicated groups. **e-j**. Summary of serum BUN (e), creatinine (f), LDH (g), AST (h), glucose (i) and lactic acid (j) level measured in the indicated mice. **k.** Representative H&E-stained, Sirius Red-stained and Masson's Trichrome-stained kidney sections from indicated mice.

Significance in survival curve was calculated using the log-rank (Mantel-Cox) test. **p*<0.05, ***p*<0.01, ****p*<0.001, and n.s., not significant (Student *t*-test). Data are represented as mean ± SEM.

Supplementary Fig. S3

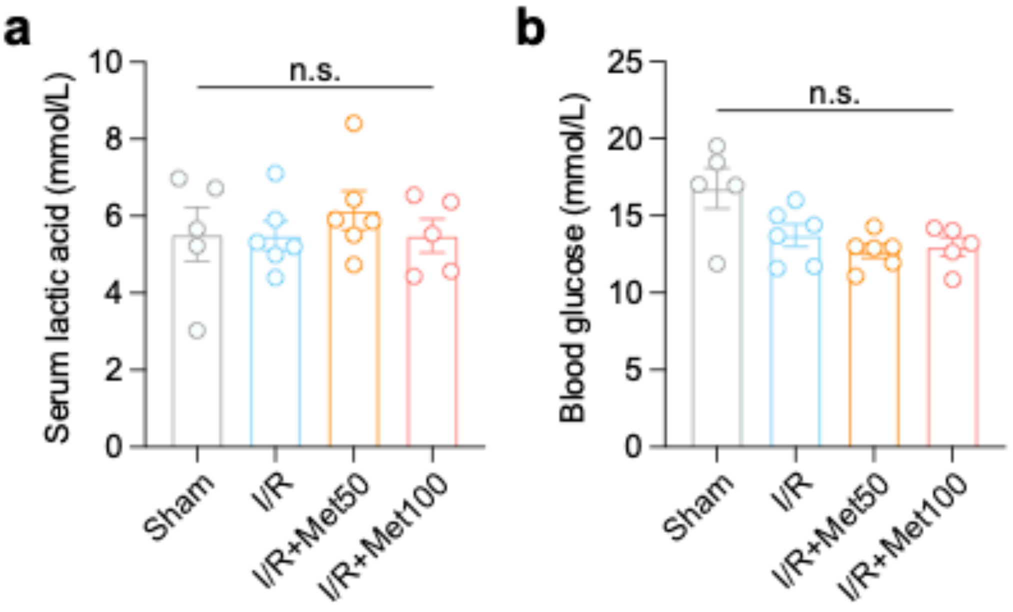

Supplementary Fig. S3 I The summary of lactic acid and glucose levels in ischemia reperfusion (I/R)-induced acute kidney injury intervened with different dose of metformin. a. Summary of the serum lactic acid level in Sham, I/R, I/R+Met50 (50 mg/kg) and I/R+Met100 (100 mg/kg) groups. **b**. Summary of the glucose level in the indicated mice.

n.s., not significant (one-way ANOVA). Data are represented as mean ± SEM.

Supplementary Fig. S4

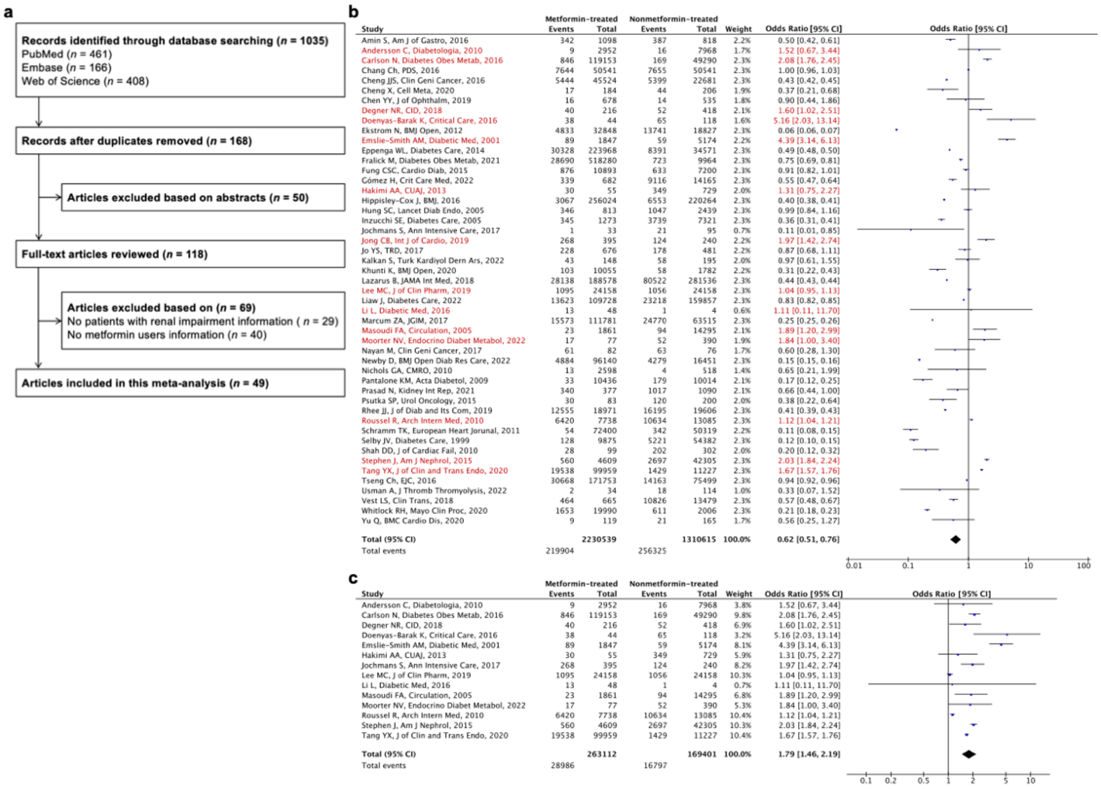

Supplementary Fig. S4 I Meta-analysis of metformin and kidney injury or diseases. a. Flow-chart depicting the literature search and selection strategy. After applying the inclusion and exclusion criteria, a total of 49 articles were included in the final meta-analysis. **b**. Forest plot showing the effect of kidney injury or diseases on the risk of metformin treatment. In this figure, the horizontal lines indicate the lower and upper limits of the 95% CI, and the size of the blue squares reflects the relative weight of each study in the meta-analysis. **c**. Summary of the above cohort studies with OR>1.

Supplementary Fig. S5

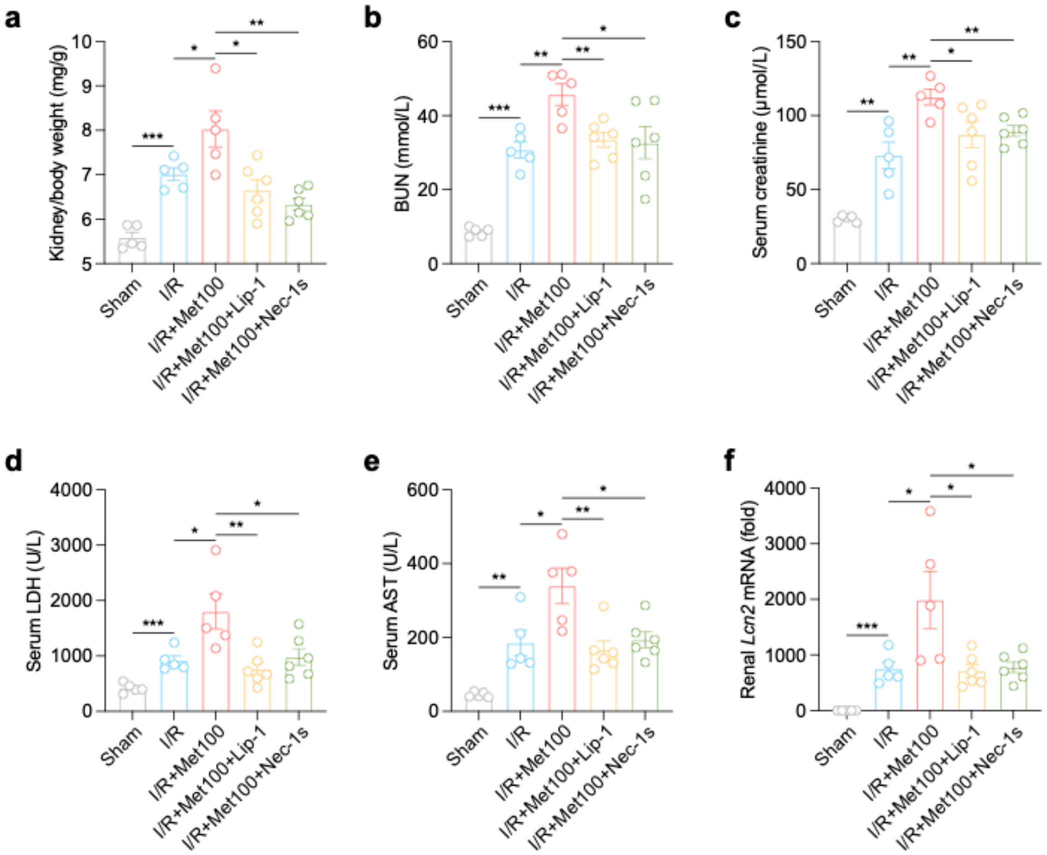

Supplementary Fig. S5 I Multiple forms of cell death contributed in metformin-aggravted I/R-induced AKI. a. Summary of the kidney to body weight ratio measured in the indicated mice; where indicated, the mice received i.p. injections of metformin (100 mg/kg), Lip-1 (1 mg/kg) and/or Nec-1s (1 mg/kg). **b-e**. Summary of serum BUN (b), creatinine (c), LDH (d), and AST (e) were measured in the indicated mice. **f**. Summary of renal *Lcn2* mRNA level measured in the indicated mice.

*$p<0.05$, **$p<0.01$, ***$p<0.001$ (one-way ANOVA). Data are represented as mean ± SEM.

Supplementary Fig. S6

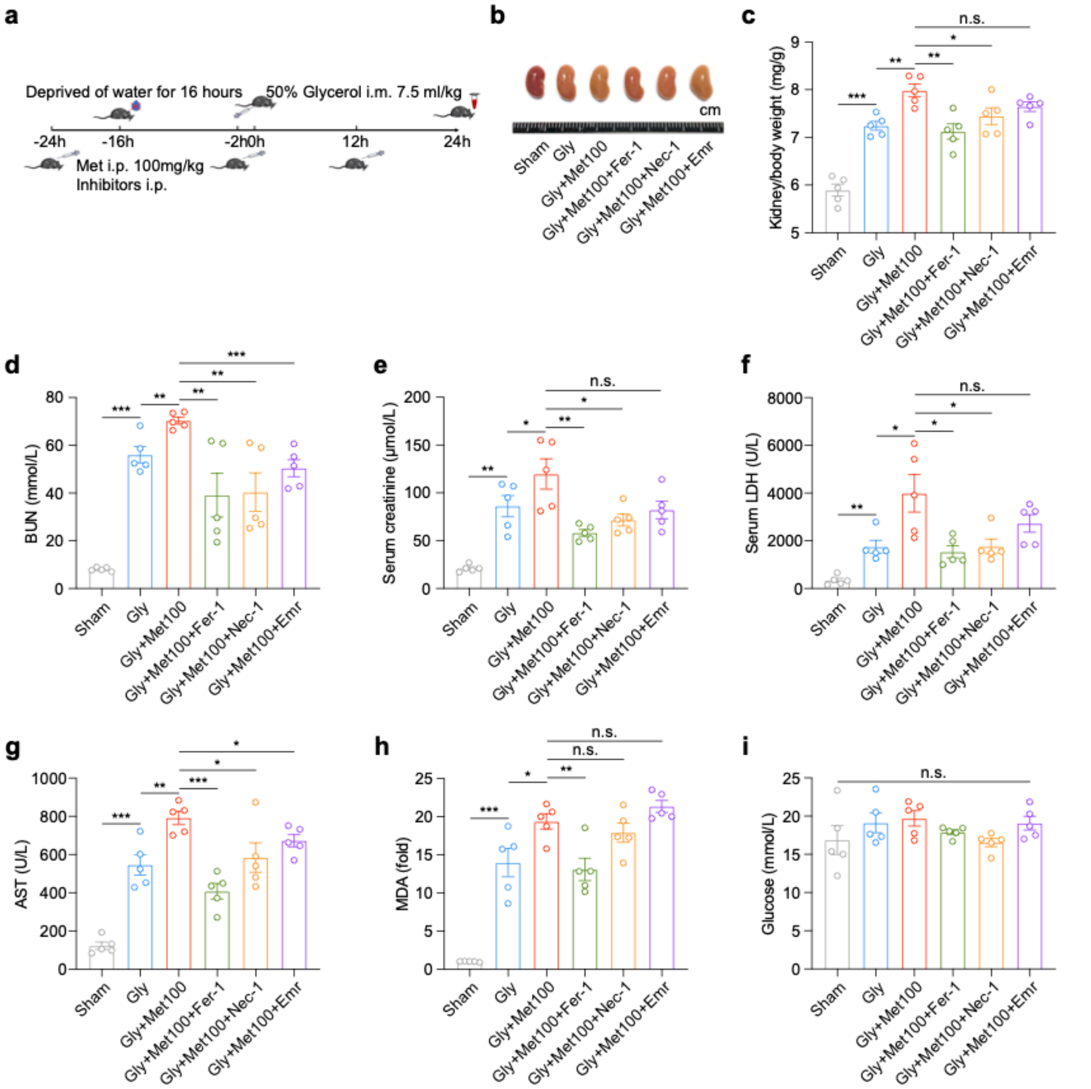

Supplementary Fig. S6 l Ferroptosis is the main form of metformin-induced renal cell death in rhabdomyolysis. a. Overview of the experimental procedure used to induce rhabdomyolysis; where indicated, the mice received an i.p. injection of metformin (100 mg/kg) and inhibitors. **b**. Representative image of kidneys from the indicated mice; where indicated, the mice received i.p. injections of metformin (100 mg/kg), Fer-1 (1 mg/kg), Nec-1 (1 mg/kg) and/or Emr (2.5 mg/kg). **c**. Summary of the kidney to body weight ratio measured in the indicated mice. **d-g**. Summary of serum BUN (d), creatinine (e), LDH (f), and AST (g) were measured in the indicated mice. **h**. Summary of renal MDA level measured in the indicated mice. **i**. Summary of serum glucose levels measured in the indicated mice. *p<0.05, **p<0.01, ***p<0.001, and n.s., not significant (one-way ANOVA). Data are represented as mean ± SEM.

Supplementary Fig. S7

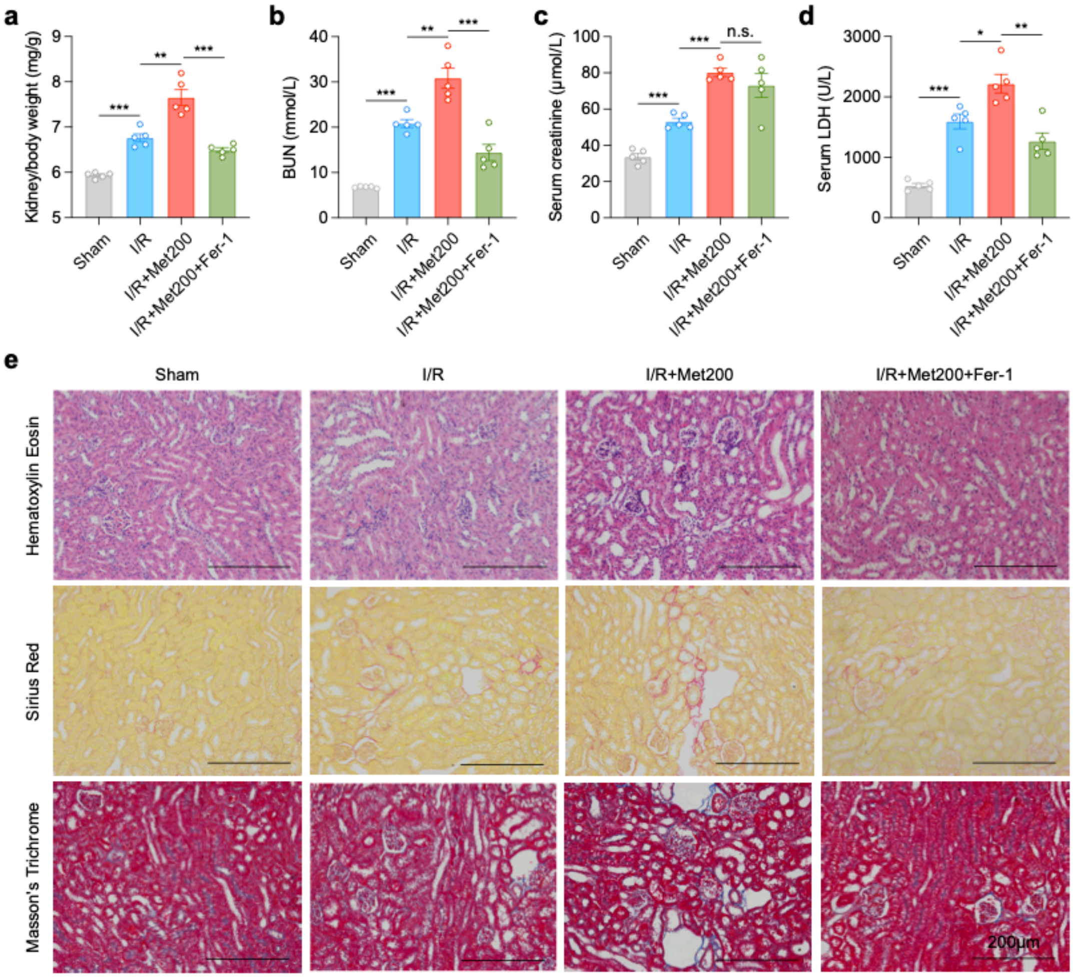

Supplementary Fig. S7 l The indexes of injury and pathological section of scRNA-seq samples. a. Summary of the kidney to body weight ratio measured in the Sham, I/R, I/R+Met200 and I/R+Met200+Fer-1 groups. **b-d**. Summary of serum BUN (b), creatinine (c), and LDH (d). measured in the indicated mice. **e**. Representative H&E-stained, Sirius Red-stained and Masson-s Trichrome-stained kidney sections from indicated mice. **p*<0.05, ***p*<0.01, ****p*<0.001, and n.s., not significant (one-way ANOVA). Data are represented as mean ± SEM.

Supplementary Fig. S8

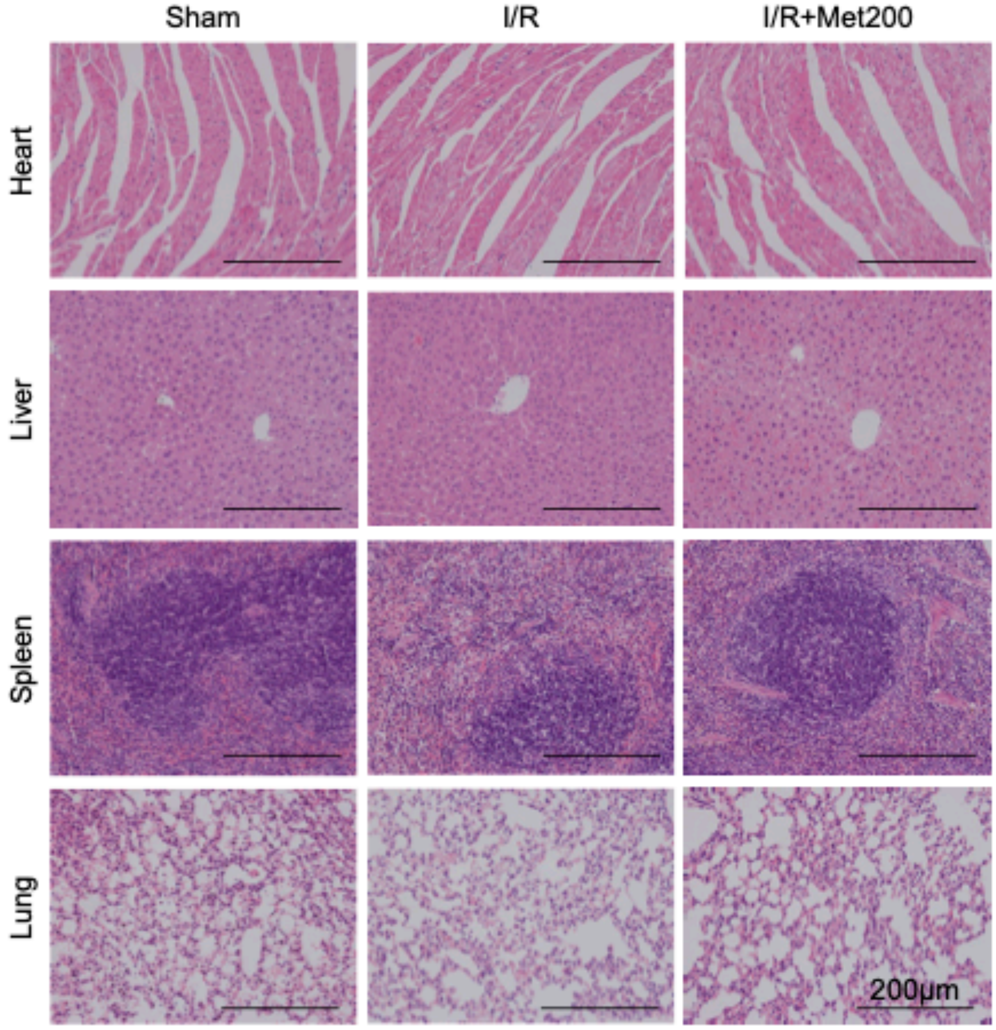

Supplementary Fig. S8 l The representative images of pathological section of heart, liver, spleen and lung in sham, I/R and I/R+Met200 groups. Representative image of hearts, livers, spleens and lungs from sham, I/R, and I/R+Met200 groups.

Supplementary Fig. S9

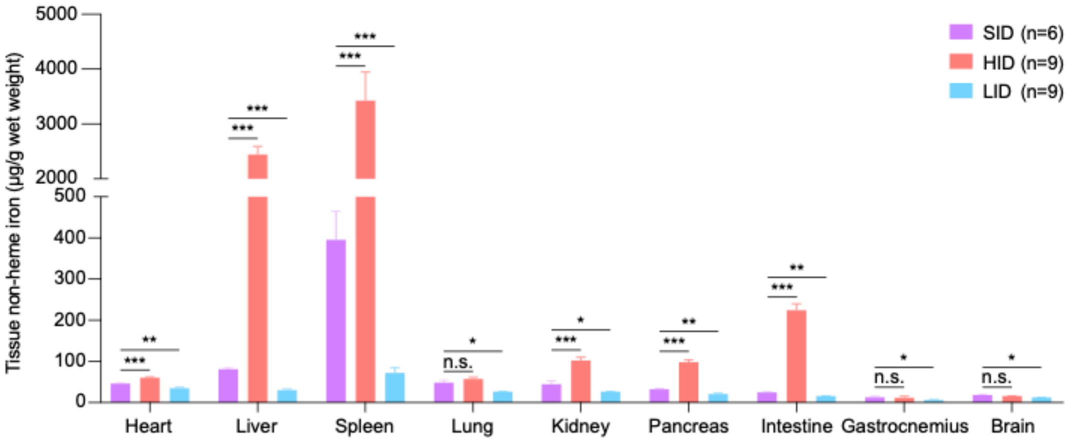

Supplementary Fig. S9 | Summary of tissue non-heme iron levels. Non-heme iron levels were measured in the indicated tissues in mice fed a standard-iron diet (SID; n=6), high-iron diet (HID; n=9), or low-iron diet (LID; n=9), followed by I/R+Met. *p<0.05, **p<0.01, ***p<0.001, and n.s., not significant (one-way ANOVA). Data are represented as mean ± SEM.

Supplementary Fig. S10

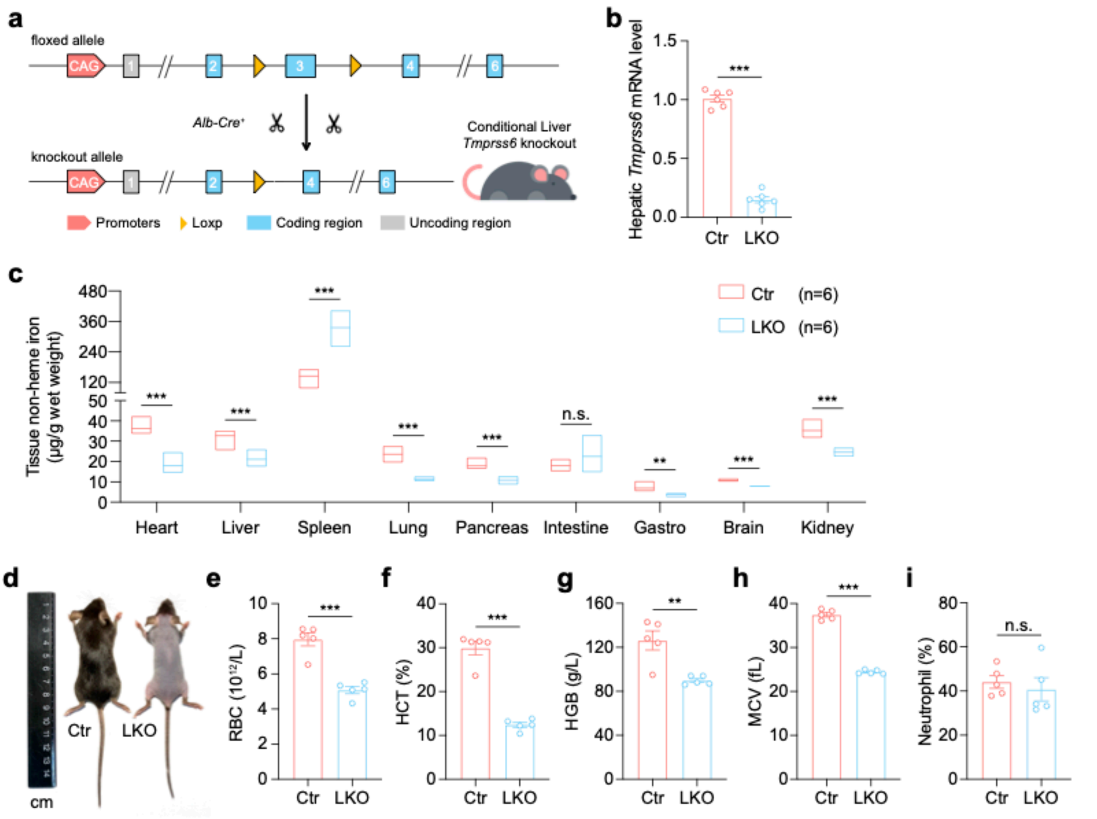

Supplementary Fig. S10 l Generation and characterization of liver-specific *Tmprss6* knockout mice. a. Schematic diagram depicting the strategy used to generate a liver-specific *Tmprss6* knockout mouse. *Tmprss6*$^{flox/flox}$ mice were crossed with *Alb-Cre* transgenic mice to generate *Tmprss6*$^{Alb/Alb}$ mice. **b**. Hepatic *Tmprss6* mRNA was measured in control (Ctr, *Tmprss6*$^{flox/flox}$) mice and in liver-specific *Tmprss6* knockout (LKO, *Tmprss6*$^{Alb/Alb}$) mice, expressed relative to control. **c**. Summary of non-heme iron levels measured in the indicated tissues of the indicated mice (n=6 per group); shown at the right are the detailed data for non-heme iron levels measured in the kidney. **d**. Representative images of adult *Tmprss6*$^{flox/flox}$ and *Tmprss6*$^{Alb/Alb}$ mice. **e-h**. Summary of the red blood cell count (e), hematocrit value (f), hemoglobin concentration (g), and mean corpuscular volume (h) measured in the indicated mice (n=5 per group). **i**. Summary of the percentage of neutrophils in the circulation of the indicated mice (n=5 per group).

**$p<0.01$, ***$p<0.001$, and n.s., not significant (Student's *t*-test). Data are represented as mean ± SEM.

Supplementary Fig. S11

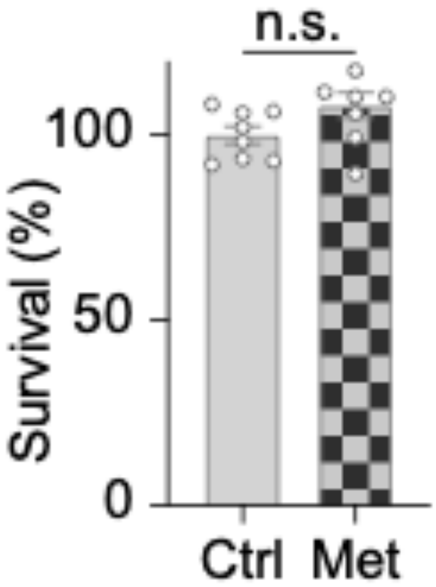

Supplementary Fig. S11 l Metformin has no toxicity on primary mouse renal tubular cells. Survival of the metformin (200 μM) treated primary mouse renal tubular cells for 24h.

n.s., not significant (Student's *t*-test). Data are represented as mean ± SEM.

Supplementary Fig. S12

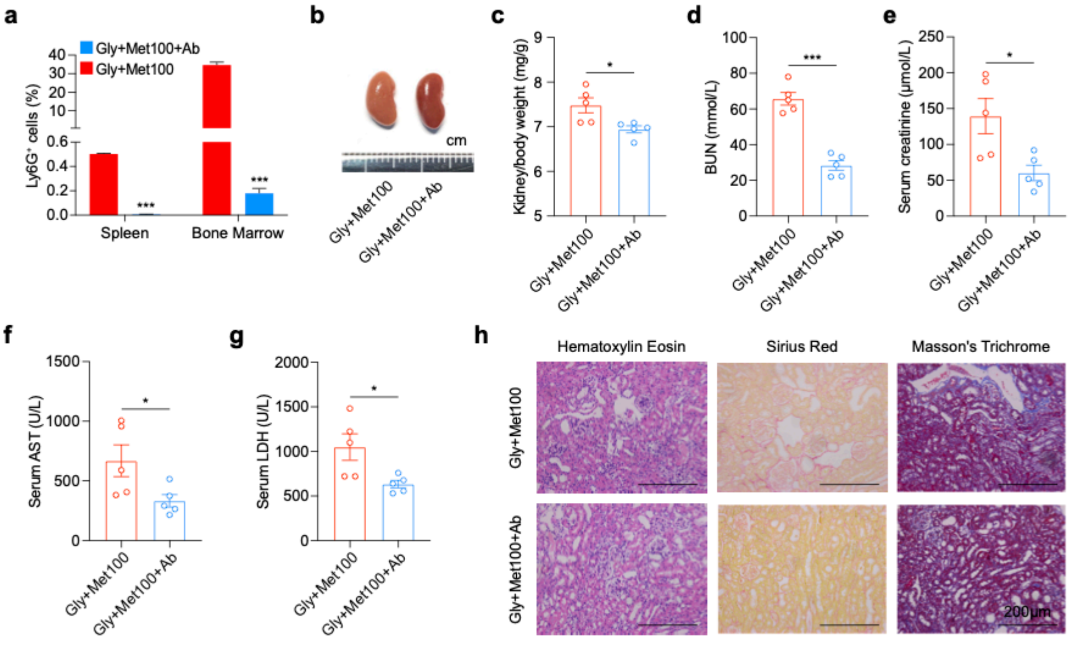

Supplementary Fig. S12 | Neutrophil clearance protects against metformin-exacerbated rhabdomyolytic acute kidney injury. a. Flow cytometry analysis of the percentage of neutrophils measured in the spleen and bone marrow of the indicated mice; where indicated, metformin and the anti-Ly6G antibody were administered by i.p. injection at 100 mg/kg and 200 μg per mouse, respectively. **b**. Representative image of kidneys in the indicated groups. **c**. Summary of the kidney to body weight ratio measured in the indicated mice. **d-g**. Summary of serum BUN (d), creatinine (e), AST (f), and LDH (g) measured in the indicated mice. **h**. Representative H&E-stained, Sirius Red-stained, and Masson's Trichrome-stained kidney sections from the indicated mice.

*p<0.05, ***p<0.001, and n.s., not significant (Student's t-test). Data are represented as mean ± SEM.

Supplementary Fig. S13

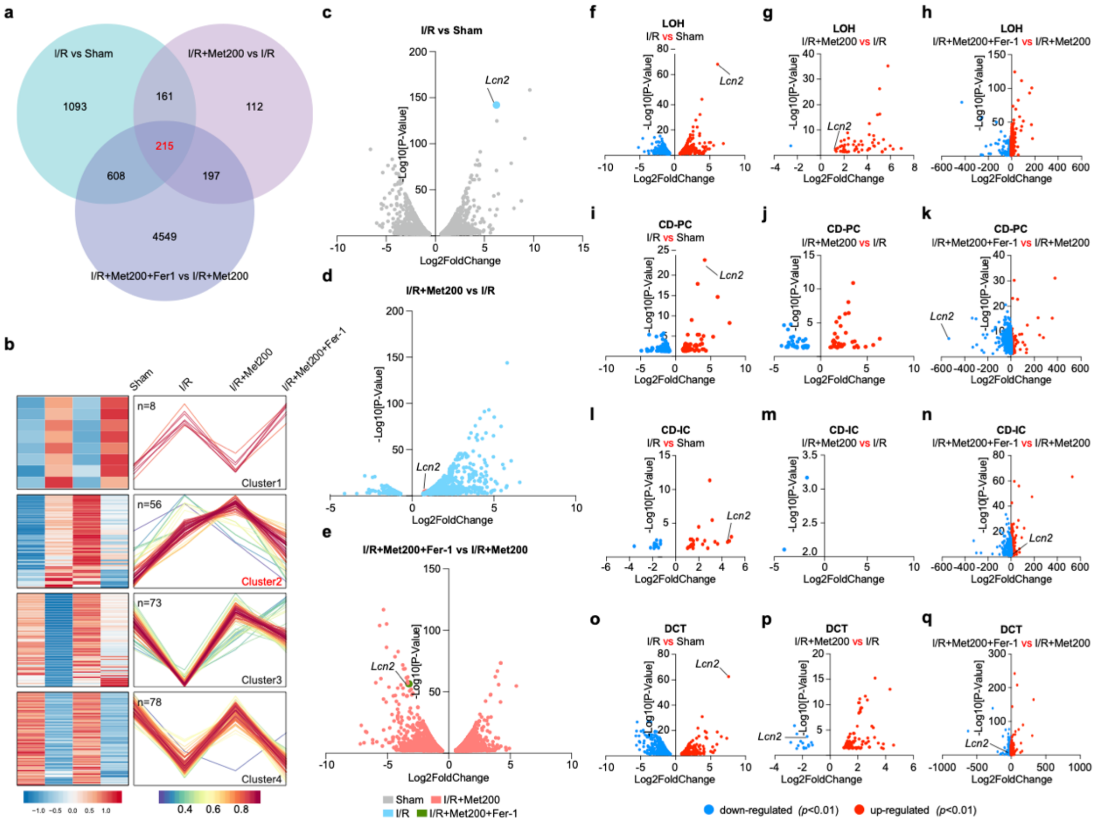

Supplementary Fig. S13 I Heatmap and volcano plot showing significantly differentially expressed genes in the indicated conditions. a. Venn diagram of overlapping significantly changed genes (p<0.001) found in each group. **b**. The genes changed in common from **Supplementary Figure S10A** are divided into 4 clusters according to the change trend via fuzzy clustering. **c-e**. Volcano plot showing significantly changed genes (p<0.001) of renal parenchymal cells in I/R vs Sham group (c), I/R+Met200 vs I/R group (d), and I/R+Met200+Fer-1 vs I/R+Met200 group (e). **f-q**. Differentially expressed genes (either up-regulated or down-regulated are shown for cells in the Loop of Henle (LOH; f-h), principal cells in the collecting duct (CD-PC; i-k), intercalated cells in the collecting duct (CD-IC; l-n), and cells in the distal convoluted tubule (DCT; o-q). Where indicated, the *Lcn2* gene is shown.

Supplementary Fig. S14

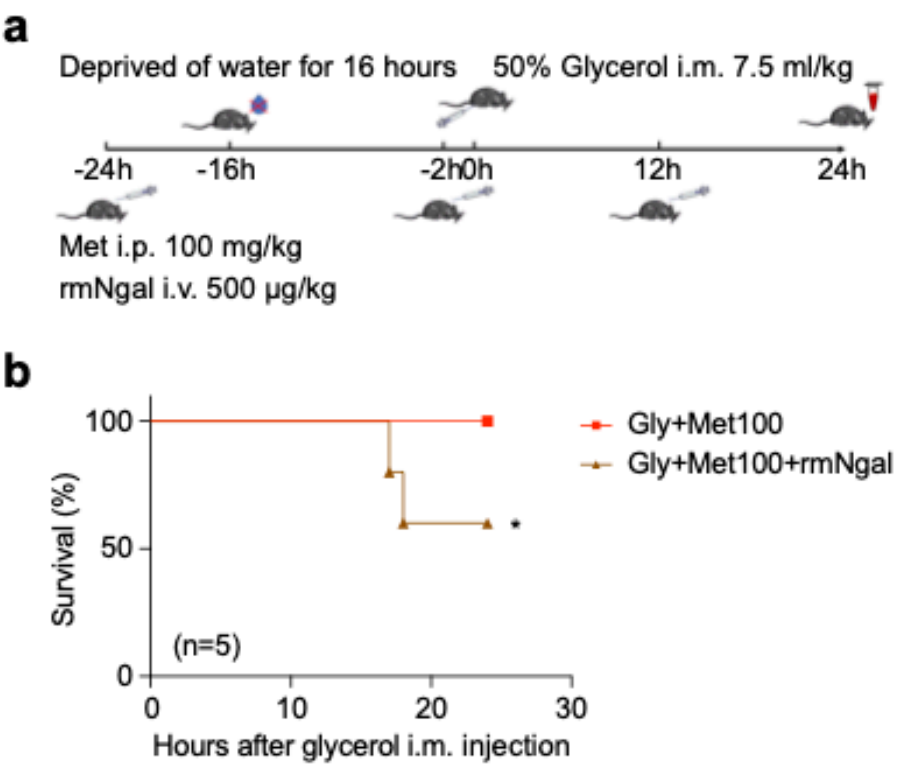

Supplementary Fig. S14 I Injection of recombinant mouse Ngal (rmNgal) causes increased mortality in rhabdomyolysis-induced mice. a. Overview of the experimental procedure for inducing rhabdomyolysis; where indicated, metformin (100 mg/kg, i.p.) and rmNgal (500 µg/kg, i.v.) were administered. **b**. Kaplan-Meier survival curves of mice in the indicated groups (n=5 per group). Significance in survival curve was calculated using the log-rank (Mantel-Cox) test.

Supplementary Fig. S15

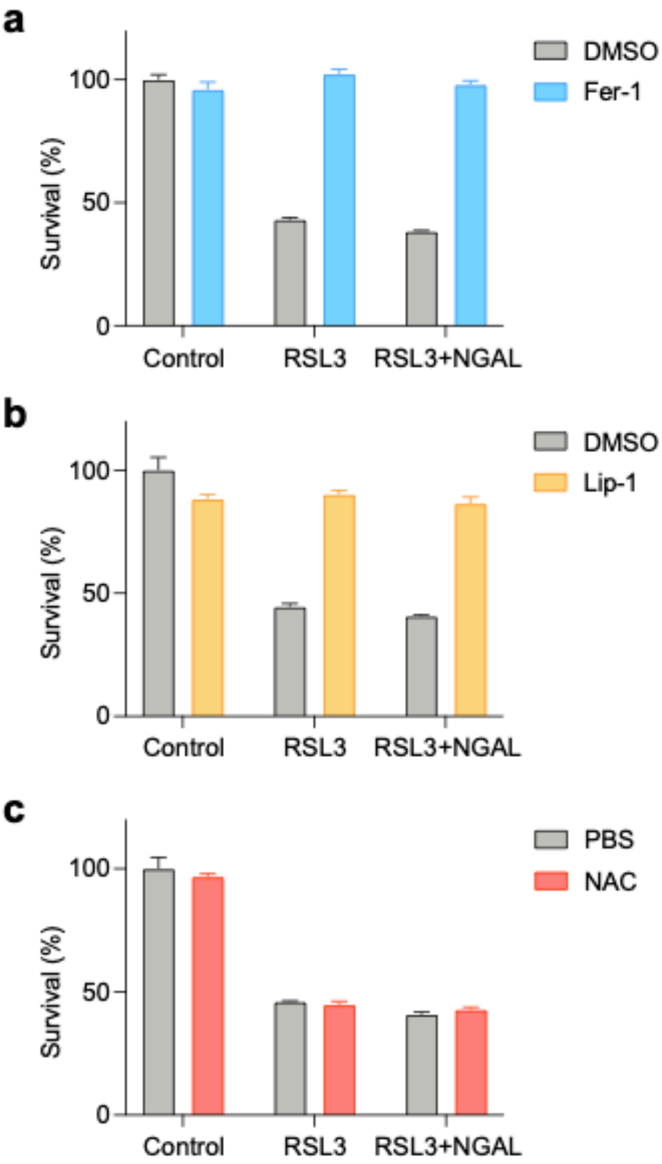

Supplementary Fig. S15 I NGAL protein has no obvious effect on ferroptotic cells. The NGAL protein used on RSL3 induced ferroptosis with Fer-1 (a), Lip-1 (b), or NAC (c).

Supplementary Fig. S16

a

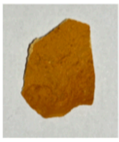

b

Sample	Fe[Metformin]$_3$·4H$_2$O	Fe[Metformin]$_3$
C (%) 28.17	28.13	32.73
N (%) 41.03	41.02	47.73
H (%) 7.24	7.42	6.87
Fe (%) 23.56	10.94	12.68
O (%)	12.50	0

c

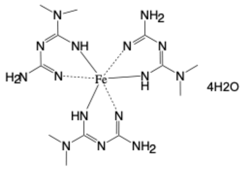

d

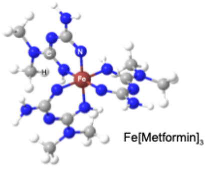

e

Index	Residue	AA	Distance (Å)	Distance H-A (Å)	Distance D-A (Å)	Interaction Type
1	79A	Trp		3.04	4.02	Hygrophobic Interactions
2	81A	Arg		2.31	3.00	
3	106A	Tyr		2.44	3.02	
4	106A	Tyr		3.21	3.80	
5	125A	Lys		2.33	3.30	
6	134A	Lys		2.63	3.57	
7	79A	Trp	4.03			π-Cation Interactions

Energy: -5.51 kcal/mol

f **g**

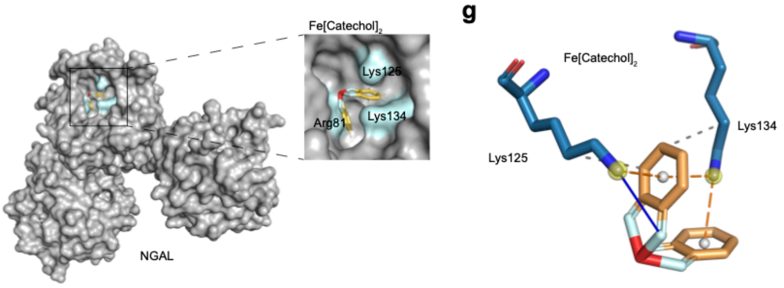

h

Index	Residue	AA	Distance (Å)	Distance H-A (Å)	Distance D-A (Å)	Interaction Type
1	125A	Lys	3.88			Hygrophobic Interactions
2	134A	Lys	3.50			
3	125A	Lys		2.83	3.70	Hydrogen Bonds
6	125A	Lys	3.15			π-Cation Interactions
7	134A	Lys	3.19			
8	134A	Lys	3.15			

Energy: -2.83 kcal/mol

Supplementary Fig. S16 l Close-up view of the electrostatic surface of the three-dimensional diagram and the interactions within the complexes. a. The appearance of the Fe[Metformin]$_3$ • 4H$_2$O complex. **b**. Element analysis of the Fe[Metformin]$_3$ • 4H$_2$O complex. **c-d**. Two-dimensional (c) and three-dimensional (d) chemical structures of the complex formed between Fe and [Metformin]$_3$ coordinated with 4H$_2$O. **e**. Types of interaction force between the Ngal protein and either Fe[Metformin]$_3$ • 4H$_2$O complex. **f**. Close-up view of the electrostatic surface of the three-dimensional structure consisting of the Ngal protein, catechol and Fe. **g**. Forces between the indicated residues in the Ngal protein and the Fe[Catechol]$_2$ complex are shown. **h**. Types of interaction force between the Ngal protein and either Fe[Catechol]$_2$.

Supplementary Fig. S17

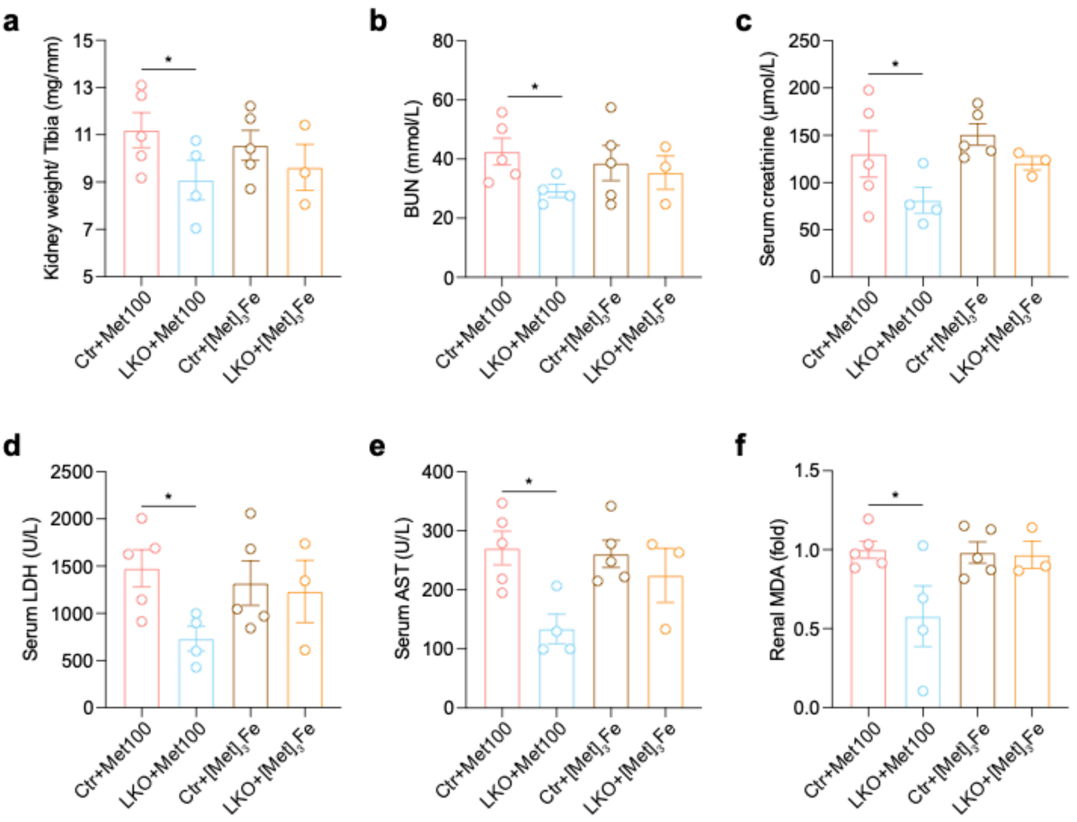

Supplementary Fig. S17 | [Metformin]$_3$Fe complex reverses the phenotype of *Tmprss6* liver specific knockout mice (LKO). a. Summary of the ratio between kidney weight and tibia length measured in the control or LKO mice with metformin or [Metformin]$_3$Fe complex. **b-e**. Summary of the serum BUN (b), creatinine (c), LDH (d), and AST (e) measured in the indicated mice. F. Summary of the renal MDA level measured in the indicated mice.

*p<0.05 (Student's t-test). Data are represented as mean ± SEM.

Supplementary Fig. S18

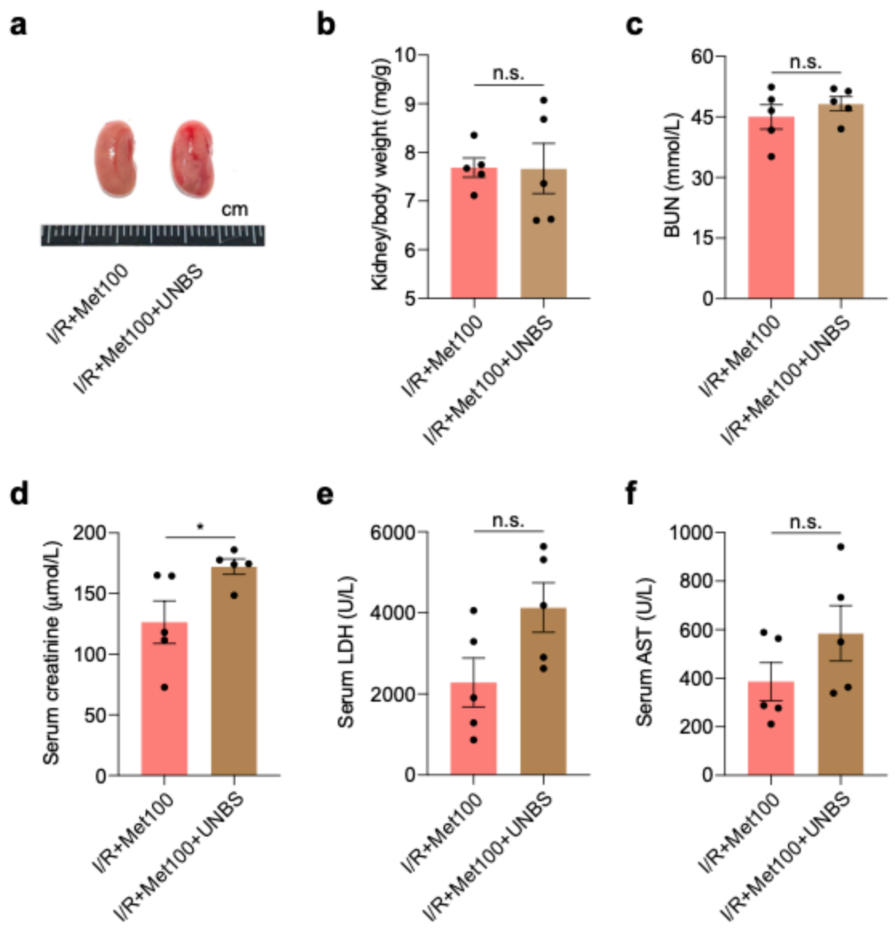

Supplementary Fig. S18 l Cxcl family inhibitor has no apparent effect on metformin nephrotoxicity. a. Representative image of kidneys removed from a mouse following I/R+Met100 (100 mg/kg), and a mouse in I/R+Met100+UNBS (UNBS5162, 50 mg/kg) group. **b**. Summary of the kidney to body weight ratio measured in the indicated mice. **c-f**. Summary of serum BUN (c), creatinine (d), LDH (e), and AST (f) measured in the indicated mice. *p<0.05 (Student's t-test). Data are represented as mean ± SEM.

Supplementary Fig. S19

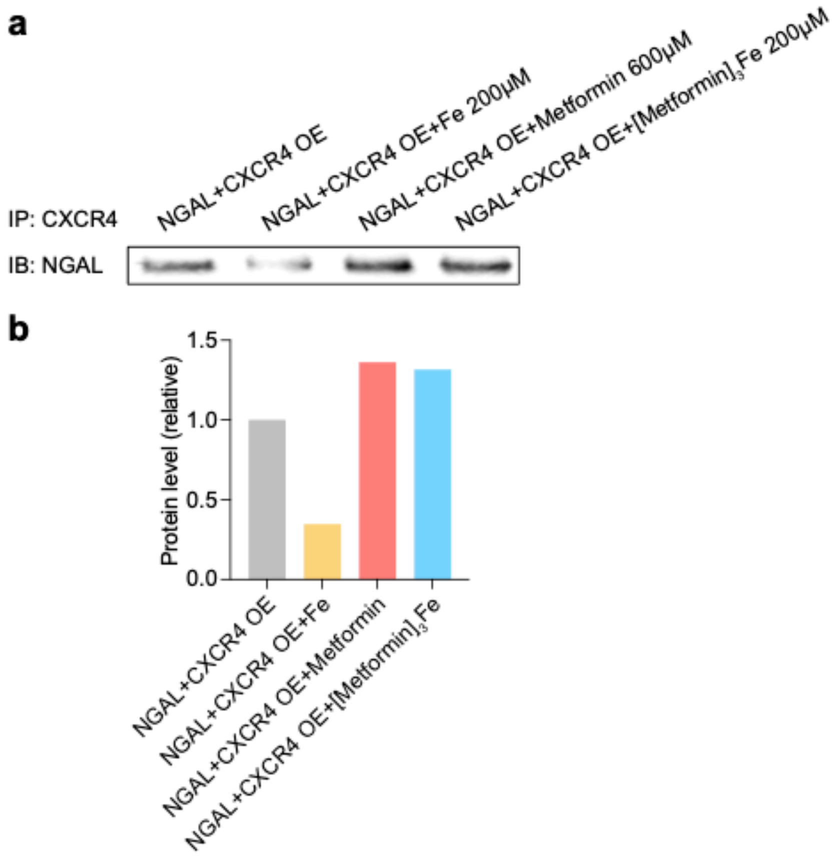

Supplementary Fig. S19 I CXCR4 and NGAL were co-expressed with $FeCl_3$ or metformin or $[Metformin]_3Fe$ complex in HEK293T cells. a. Co-IP of NGAL and CXCR4 under indicated conditions. After 48 hours transfection, the cell lysates were immunoprecipitated using protein A/G beads to pull-down CXCR4 (IP), respectively, followed by immunoblotting (IB) with the NGAL antibody. **b**. Relative quantification of immunoblots.

Supplementary Table S1.

Characteristics of the 49 studies included in the meta-analysis.

NO.	First author, source year	Total cases	Age in years, mean ± SD or median (range)	Standard	Metformin-treated, n	Non-metformin-treated, n	Renal impairment (Met), n	Renal impairment (Non-Met), n
1	Amin S, Am J of Gastro, 2016	1916	NA	CKD	1098	818	342	387
2	Andersson C, Diabetologia, 2010	10920	NA	NA	2952	7968	9	16
3	Carlson N, Diabetes Obes Metab, 2016	168443	NA	eGFR < 60ml/min/1.73m^2	119153	49290	846	169
4	Chang Ch, PDS, 2016	101082	NA	CKD + RF	50541	50541	7644	7655
5	Chen YY, J of Ophthalm, 2019	68205	56.1 ± 12.6	CKD	45524	22681	5444	5399
6	Cheng JJS, Clin Geni Cancer, 2016	390	NA	Chronic renal failure	184	206	17	44
7	Cheng X, Cell Meta, 2020	1213	63.0 (56.0-69.0)	CKD	678	535	16	14
8	Degner NR, CID, 2018	634	71.0 (57.5-79.2)	CKD	216	418	40	52

9	Doenyas-Barak K, Critical Care, 2016	162	NA	AKI	44	118	38	65
10	Ekstrom N, BMJ Open, 2012	51675	65.3 ± 9.8	eGFR < 60ml/min/1.73m^2	32848	18827	4833	13741
11	Emslie-Smith AM, Diabetic Med, 2001	7021	63	serum creatinine ≥ 150 mmol/l	1847	5174	89	59
12	Eppenga WL, Diabetes Care, 2014	258539	NA	eGFR < 60ml/min/1.73m^2	223968	34571	30328	8391
13	Fralick M, Diabetes Obes Metab, 2021	528244	NA	CKD + AKI +DN	518280	9964	28690	723
14	Fung CSC, Cardio Diab, 2015	18093	NA	CKD stage 3	10893	7200	876	633
15	Gómez H, Crit Care Med, 2022	14847	NA	AKI	682	14165	339	9116
16	Hakimi AA, CUAJ, 2013	784	62	High pathological grade in RCC	55	729	30	349
17	Hippisley-Cox J, BMJ, 2016	476288	NA	CKD	256024	220264	3067	6553
18	Hung SC, Lancet Diab Endo, 2005	3252	NA	Nephrologist visits > 6	813	2439	346	1047
19	Inzucchi SE, Diabetes Care, 2005	8594	NA	creatinine ≥ 1.5mg/dl	1273	7321	345	3739
20	Jo YS, TRD, 2017	128	NA	CKD	33	95	1	21
21	Jochmans S, Ann Intensive Care, 2017	635	NA	AKI	395	240	268	124
22	Jong CB, Int J of Cardio, 2019	1157	NA	CKD	676	481	228	178

23	Kalkan S, Turk Kardiyol Dern Ars, 2022	343	NA	AKI	148	195	43	58
24	Khunti K, BMJ Open, 2020	11837	56.5 ± 11.7	eGFR < 60ml/min/1.73m^2	10055	1782	103	58
25	Lazarus B, JAMA Int Med, 2018	470114	60.4 ± 15.5	eGFR < 60ml/min/1.73m2	188578	281536	28138	80522
26	Lee MC, J of Clin Pharm, 2019	48316	NA	CKD	24158	24158	1095	1056
27	Li L, Diabetic Med, 2016	52	NA	eGFR < 60ml/min/1.73m^2	48	4	13	1
28	Liaw J, Diabetes Care, 2022	269585	NA	DN	109728	159857	13623	23218
29	Marcum ZA, JGIM, 2017	175296	NA	eGFR < 60ml/min/1.73m^2	111781	63515	15573	24770
30	Masoudi FA, Circulation, 2005	16156	NA	creatinine ≥ 133 umol/L	1861	14295	23	94
31	Moorter NV, Endocrino Diabet Metabol, 2022	467	NA	CKD	77	390	17	52
32	Nayan M, Clin Geni Cancer, 2017	158	NA	eGFR < 90ml/min/1.73m^2	82	76	61	63
33	Newby D, BMJ Open Diab Res Care, 2022	112591	NA	eGFR < 60ml/min/1.73m2	96140	16451	4884	4279
34	Nichols GA, CMRO, 2010	3116	NA	Nephropathy	2598	518	13	4
35	Pantalone KM, Acta Diabetol, 2009	20450	NA	Renal disease	10436	10014	33	179

36	Prasad N, Kidney Int Rep, 2021	1467	NA	CKD stage 3 4	377	1090	340	1017
37	Psutka SP, Urol Oncology, 2015	283	NA	eGFR < 60ml/min/1.73m^2	83	200	30	120
38	Rhee JJ, J of Diab and Its Com, 2019	38577	72.0 ± 10.6	eGFR < 60ml/min/1.73m^2	18971	19606	12555	16195
39	Roussel R, Arch Intern Med, 2010	20823	NA	eGFR < 60ml/min/1.73m^2	7738	13085	6420	10634
40	Schramm TK, European Heart Jorunal, 2011	122719	NA	CKD + AKI	72400	50319	54	342
41	Selby JV, Diabetes Care, 1999	64257	NA	plasma creatinine > 1.5mg/dl	9875	54382	128	5221
42	Shah DD, J of Cardiac Fail, 2010	401	56 ± 11	CKD stage 3 4 5	99	302	28	202
43	Stephen J, Am J Nephrol, 2015	46914	NA	CKD	4609	42305	560	2697
44	Tang YX, J of Clin and Trans Endo, 2020	111186	66.37 ± 12.25	CKD (New/Ongoing Metformin)	99959	11227	19538	1429
45	Tseng Ch, EJC, 2016	247252	NA	Nephropathy	171753	75499	30668	14163
46	Usman A, J Thromb Thromyolysis, 2022	148	NA	Renal disease	34	114	2	18
47	Vest LS, Clin Trans, 2018	14144	NA	eGFR < 60ml/min/1.73m^2	665	13479	464	10826

48	Whitlock RH, Mayo Clin Proc, 2020	21996	NA	eGFR < 60ml/min/1.73m^2	19990	2006	1653	611
49	Yu Q, BMC Cardio Dis, 2020	284	NA	eGFR < 60ml/min/1.73m^2	119	165	9	21

Supplementary Table S2.

The number of each cell type in different treatment groups.

		Sham	I/R	I/R+Met200	I/R+Met200+Fer-1
Nephron	Podo	10	24	4	11
	PT	26894	20239	13918	15781
	LOH	902	1322	379	429
	DCT	913	1781	618	1666
Ureteric epithelium	CD-PC	179	373	71	166
	CD-IC	164	196	140	327
	CD-Trans	30	79	2	130
Immune cells	Neutro	139	200	1152	94
	Macro	357	396	225	119
	T Lymph	188	177	105	82
	B Lymph	45	42	64	33
	NK	208	194	120	100
Interstitial cells	Endo	613	3653	2502	2083
	Fib	86	342	178	91
